# Neural basis of the interaction between alerting and executive control

**DOI:** 10.3389/fpsyg.2025.1672530

**Published:** 2025-10-24

**Authors:** Filip Kottik, Bartłomiej Panek, Ilona Kotlewska, Mikołaj Compa, Dariusz Asanowicz

**Affiliations:** ^1^Institute of Psychology, Jagiellonian University, Krakow, Poland; ^2^Doctoral School of Social Sciences, Jagiellonian University, Krakow, Poland

**Keywords:** alerting, conflict, executive control, conflict-related theta, midfrontal theta, attentional networks, attention network test (ANT)

## Abstract

Attentional alerting—evoked by an accessory stimulus such as a tone presented briefly before target onset—generally decreases response time (RT) but this decrease is smaller in trials with a conflict (induced, e.g., by presenting flankers that are incongruent with the target stimulus). This somewhat paradoxical interaction is usually interpreted as an increased conflict cost, possibly indicating less efficient conflict resolution. The present study investigated the electrophysiological activity underlying the impact of alerting on response conflict processing. Human participants performed a modified version of the Eriksen flanker task while EEG was recorded. Alerting tone was presented either with a short stimulus-onset asynchrony (SOA 100 ms) or long (SOA 800 ms), or was not presented at all (no alerting condition). To examine how alerting modulates motor, visual, and central executive processing, we analyzed evoked (i.e., phase-locked) activity (event-related potentials or ERPs), induced (i.e., non-phase-locked) activity (local power modulations), and phase coherence-based functional connectivity. Time-frequency power and phase of the EEG signal were measured from EEG sources isolated with a method employing the generalized eigenvalue decomposition (GED). Behavioral results replicated the alerting-conflict interaction in RT (but not in error rates). In the EEG results, effects of alerting were observed as: (i) an increase in conflict-related midfrontal theta power, (ii) a decrease in midfrontal N2 amplitude, (iii) a decrease in LRP latency, and (iv) an increase in N2pc amplitude. Moreover, several alerting effects were present only in the SOA 800 condition, suggesting that they may be specific to endogenous alertness: (i) a suppression of the flanker effect on response-related lateralization of alpha/mu power, (ii) an increase in the flanker effect on LRP latency, and (iii) an increase in the flanker effect on the target-related contralateral suppression of visual alpha power. The findings suggest that alerting dynamically modulates both the emergence and resolution of response conflict through widespread changes within a neural network that can be characterized as a “*selection-for-action”* system. Alerting may serve as a key modulator of neural dynamics in this system.

## 1 Introduction

The aim of this study was to examine the local and inter-regional neural activity underlying the interaction between attentional alerting and response conflict processing.

### 1.1 Alertness

Alertness is a function of the human attention system that facilitates achieving a state of readiness to process and respond to external events (Posner, 2008; Posner and Boies, 1971). It enables efficient interactions with the environment. Lapses in alertness—be it due to temporal fluctuations of its capacity or lack of alerting stimulation—may hinder our cognitive and behavioral efficiency. For instance, inattentive clinicians commit medical errors, and unfocused drivers cause road accidents, both often resulting in injuries or death. On the other hand, in line with the classic Yerkes-Dodson law, some tasks are performed better at lower levels of alertness, and alerting may even impair their completion. It is imperative to understand the cognitive and neural mechanisms of the alerting process and its interactions with other sensorimotor functions underlying our cognition and action (cf. e.g., Hackley, 2009; Poth, 2025; Tang et al., 2012).

A large number of studies have shown improvements in behavioral performance when a short-lived phasic alerting was induced by presenting a visual or auditory accessory stimulus within about 500 ms before the onset of a target stimulus (for a review see, e.g., Hackley, 2009; Posner, 2008; Poth, 2025). The improvement is usually reflected in faster response times (RT). Electrophysiological and imaging studies have demonstrated that this behavioral enhancement is produced by increasing the speed of processing and/or lowering the “decision threshold” at several stages of the sensorimotor pathway, from the initial perceptual processing and stimulus discrimination (Böckler et al., 2011; Fischer et al., 2012; Kusnir et al., 2011; Petersen et al., 2017; Wiegand et al., 2017) to decision making and early phases of response selection and activation (Böckler et al., 2011; Hackley and Valle-Inclán, 1998, 1999; Hackley et al., 2009; Yanaka et al., 2010; Yoshida et al., 2013). Imaging studies have also showed that the alerting process relies on activation of a distributed network of anterior, posterior, and subcortical areas characterized as an alerting network (Fan et al., 2005; Sadaghiani and D'Esposito, 2015; Xuan et al., 2016; for a review see, e.g., Posner and Rothbart, 2007; Petersen and Posner, 2012).

### 1.2 Interaction between alerting and conflict

Negative effects of alerting have also been shown in experimental studies. When participants perform tasks requiring resolution of conflict between two or more manual responses, such as the flanker task and Simon task (for a review of these tasks see, e.g., Egner, 2008), phasic alerting often increases behavioral costs of the conflict. In the flanker task (Eriksen and Eriksen, 1974; Ridderinkhof et al., 2021) participants respond to a target stimulus (e.g., a letter or an arrow), usually with a left or right button press. The central target is surrounded by flanker stimuli, which may signal the same response as the target (congruent condition) or the opposite response (incongruent condition). In the latter case, a response conflict is induced by simultaneous activation of two competing response programs. The required response is activated based on a task-relevant stimulus (target), while task-irrelevant stimuli (flankers) automatically trigger the competing incorrect response (see, e.g., Grent-‘t-Jong et al., 2013). Behavioral responses are therefore slower and more error prone in the incongruent than in congruent trials, which is referred to as the congruency or flanker effect. Resolving this conflict is thought to involve executive control over response selection and execution (see, e.g., Fan et al., 2002; Klein et al., 2014; Nigbur et al., 2012; Verleger et al., 2009). In the Posner’s view, executive control is one of the functions of the attention system, carried out by an executive network (Klein, 2022; Posner and Rothbart, 2007). Getting back to the main issue; alerting typically improves RTs in both flanker conditions. However, this alerting-induced improvement is usually smaller in the incongruent condition. Thereby the scores of conflict costs (incongruent *minus* congruent condition) are larger in trials with alerting stimuli, suggesting a less efficient conflict resolution (e.g., Asanowicz and Marzecová, 2017; Callejas et al., 2004, 2005;^19,70^; Spagna et al., 2014; the interaction was also shown in a meta-analysis of studies using the attention network test [ANT] by Macleod et al., 2010).

Theoretical accounts of this somewhat paradoxical interaction between alerting and conflict processing have been varied. Three main perspectives can be distinguished here. One view proposes that alerting accelerates automatic and impulsive response selection, which facilitates activation of incorrect stimulus–response (S-R) links and promotes less controlled response execution (Fischer et al., 2012; Böckler et al., 2011). Somewhat similar is the early onset hypothesis proposing that alerting shortens stimulus-encoding and decision making, which reduces the time available for effective conflict resolution and proper response selection (Nieuwenhuis and de Kleijn, 2013; Schneider, 2018a). An alternative to the above accounts is a view that alerting enhances processing of salient events and mobilizes executive control, thereby improving the control of response inhibition and response execution (Weinbach et al., 2015). A third group of hypotheses proposes that alerting affects perceptual processing, either by widening the scope of attention and facilitating processing of spatial features (Weinbach and Henik, 2011, 2012a, 2012b) or promoting spatial grouping of visual information (Schneider, 2018b), consequently increasing the impact of incongruent flankers. Thus far, no decisive evidence has been found for any of those accounts (see, e.g., Cappucci et al., 2021; Han and Proctor, 2023; Schneider, 2019a, 2019b; Seibold, 2018). It therefore remains unclear why and how alerting increases the response conflict scores.

### 1.3 Neural basis of alerting-conflict interaction

Studies investigating the neural basis of the interaction between alerting and conflict processing have been scarce. In an fMRI study, Xuan et al. (2016) demonstrated that the interaction was related to the activation of the inferior and middle frontal gyri, anterior insula, intraparietal sulcus, and subcortical regions of putamen. The authors concluded that this may reflect shared neural resources of the alerting and executive control networks.

An EEG study by Asanowicz et al. (2019) showed that alerting may affect processing of response conflict at three stages of the S-R processing pathway. First, by facilitating the automatic processing of incongruent stimuli that increases interference within the S-R translation process (cf. the aforementioned account by Fischer et al., 2012), which was reflected in modulations of the P3b component of the event-related potential or ERP (cf. e.g., Verleger et al., 2015a, 2015b). Second, by enhancing the automatic activation of the incorrect response programs (triggered by incongruent flankers), reflected in an increased initial incorrect activation in the lateralized readiness potential (LRP, for an LRP review see, e.g., Smulders and Miller, 2012). Third, by increasing the involvement of executive control in response to the increased conflict, reflected in a larger conflict-related midfrontal theta-band power in the alerting trials, compared to non-alerting trials. The so-called conflict-theta refers to an increase of midfrontal activity in the incongruent vs. congruent trials, thought of as a reflection of oscillatory mechanism of conflict detection and resolution (for a review, see Cavanagh and Frank, 2014; Cavanagh and Cohen, 2022). Therefore, Asanowicz et al. (2019) concluded that alerting affects both the emergence of conflict and conflict control.

In a recent EEG study, Tromp et al. (2024) replicated the finding that alerting increases conflict-related midfrontal theta power—in agreement with the hypothesis that the stronger conflict in the alerting trials increases the involvement of the neural mechanism dedicated to resolving this conflict (cf. Asanowicz et al., 2019). Moreover, Tromp et al. (2024) did not replicate the interaction between alerting and conflict in the modulations of the LRP (in other words, the response-related contralateral-ipsilateral lateralization of the motor activity was not modified by alerting). However, they instead observed that alerting increased the amplitude of the overall motor activity bilaterally. Specifically, the readiness potential at both the contralateral and ipsilateral motor sites (relative to the responding hand) had larger amplitudes in the alerting trials. The authors concluded that this reflects an enhancement of an urgency signal (cf. Cisek et al., 2009), which in turn amplifies competition between evidence accumulation and shortens RTs, thereby increasing the flanker interference (cf. Van der Lubbe et al., 2025). This therefore was yet another hypothesis on the alerting-conflict interaction.

Lastly, it should be mentioned that several other ERP studies reported analysis of the alerting-conflict interaction on the ERPs but mostly with no significant interactive effects (e.g., Abundis- Gutiérrez et al., 2014; Luna et al., 2023; Neuhaus et al., 2010). Only a study by Zani and Proverbio (2017) reported findings hinting at an effect alerting on the amplitude of a conflict-related anterior negativity in the ERP. In conclusion, little is known about the neural underpinnings of the alerting-conflict interaction.

### 1.4 Present study

In the present study, following the described above lines of evidence, we investigated the electrophysiological correlates of the alerting-conflict interaction. We focused on modulations of the local and inter-areal activity related to the involvement of executive control, and to motor and visual selective processing during conflict resolution. Human participants performed a variant of the Eriksen flanker task (cf. Asanowicz et al., 2021) in which alerting tone could precede target onset (cf. Callejas et al., 2004). Behavioral responses and EEG activity were recorded during task performance. The EEG data analysis included analysis of (i) induced (i.e., time-locked but non-phase-locked) oscillatory activity, (ii) evoked (i.e., time- and phase-locked) non-oscillatory activity, i.e., the ERPs—both as assessment of local activity, and (iii) inter-areal oscillatory phase coherence as a measure of functional connectivity. To assess the induced oscillatory activity, we first isolated three groups of EEG sources in three areas: the medial prefrontal (midfrontal), left and right centro-lateral (motor), and left and right lateral occipital (visual). To this end, we used a multivariate source separation method based on the generalized eigenvalue decomposition (GED) (Cohen, 2022; de Cheveigné and Arzounian, 2015). Functionally, the isolated sources are considered here as “essential nodes” (cf. e.g., Zeki et al., 1999) underlying central executive, motor, and visual processing, respectively.

To examine how alerting affected the central executive processes controlling the resolution of response conflict (induced by the incongruent flankers), we assessed two indices of local midfrontal activity—theta-band power and the N2 component of the ERP, and inter-areal theta phase coherence as an index of functional connectivity of the midfrontal area with task-relevant motor and visual areas. Moreover, to assess possible effects of alerting on the implementations of executive control over ongoing motor processes, we analyzed the local motor-related activity, including the response related modulations of oscillatory activity (cf. e.g., Asanowicz et al., 2021; Van der Lubbe et al., 2025) and the LRPs (cf. Asanowicz et al., 2019). As mentioned above, conflict-related midfrontal theta power is thought to reflect the neurophysiological oscillatory mechanism of executive control (Cavanagh and Cohen, 2022; Cavanagh and Frank, 2014; Cohen, 2014a), and originates from the medial frontal cortex (Beldzik et al., 2022; Cohen and Ridderinkhof, 2013). The midfrontal N2 is also commonly observed to be larger in conflict than in non-conflict task conditions (Asanowicz et al., 2021; Heil et al., 2000; Kopp et al., 1996). The N2 also originates from the medial frontal cortex (including the ACC; Van Veen and Carter, 2005; Yeung et al., 2004) and may be interpreted as a signature of conflict monitoring and detection (Asanowicz et al., 2021; Cohen and Donner, 2013; Yeung et al., 2004). The medial frontal cortex has also been considered as an “executive hub” of a sensorimotor network coordinating the processes of dynamic coupling and decoupling of currently relevant perceptual and motor processes (Asanowicz et al., 2021; Cavanagh and Frank, 2014). It has been proposed that this long-range executive connectivity may be mechanistically implemented by phase-locking of theta-band oscillations in the communicating neural assemblies (cf. Siegel et al., 2012; Fries, 2005). Indeed, inter-areal theta phase coherence between the midfrontal area and task-relevant visual and motor areas has been repeatedly observed to be stronger in the conflict than in non-conflict conditions (e.g., Asanowicz et al., 2021, 2023; Van der Lubbe et al., 2025; for review see Cavanagh and Cohen, 2022; Cavanagh and Frank, 2014). Accordingly, we expected that if alerting directly affects the process of central executive control, this should be reflected in modulations of both the local and inter-areal midfrontal activity, consistently with the alerting-conflict interaction in the behavioral measures.

Additionally, we added two modifications to the flanker task procedure. First, we used a bilateral visual stimulus array so that we could isolate the lateralized EEG activity (contralateral vs. ipsilateral, relative to the target visual hemifield) related to visuospatial selection. In detail, a target stimulus was presented in either the left or right hemifield within a vertical array of flankers (either congruent or incongruent), while a set of neutral distractors was simultaneously displayed in the opposite hemifield (cf. Asanowicz et al., 2021; Evert et al., 2003). The rationale for this was that one of the aforementioned accounts of the alerting-conflict interaction predicts the neural locus of the interaction in the visual processing (Schneider, 2018b), and particularly in visuospatial selection (Weinbach and Henik, 2011, 2012a, 2012b), whereas Asanowicz et al. (2019) observed the EEG correlates of the interaction on several stages of processing but not in the visual processing. A reason for the lack of an effect at the visual level may be that the procedure was not adequate to capture the effects of visuospatial selection. Here, we aimed to address this issue by examining the target selection-related lateralized activity in the alpha band (Asanowicz et al., 2021; Bacigalupo and Luck, 2019), and the N2pc component of the ERP interpreted as the signature of stimulus selection (Constant et al., 2025; Eimer, 1996). If alerting amplifies visual selection, this amplification was expected to be reflected in a stronger selection-related lateralization of the visual activity.

The second modification was added to examine the time-course of alerting. Following the study by Asanowicz and Marzecová (2017), we introduced two stimulus-onset asynchrony (SOA) intervals between alerting tone and target onsets: 100 and 800 ms. As the tone is assumed to initially evoke a short-lived exogenous phasic alerting, the SOA 100 condition was assumed to capture immediate effects of phasic alerting on conflict processing. Whereas in the long SOA condition, at the time-point of target onset the exogenous activity evoked by phasic alerting is assumed to be already (mostly) decayed, and endogenous alertness is expected to be developed instead (cf. Fernandez-Duque and Posner, 2001; Klein and Ivanoff, 2011). The latter is assumed to be an effect of a slower but more sustained endogenous increase in expectancy and readiness, which allows for better preparation for processing and responding to the expected stimulus (cf. Fan et al., 2007; Hackley et al., 2009; Périn et al., 2010; Posner, 2008; Weinbach and Henik, 2013). Thus, with the long SOA, the efficiency of conflict processing was expected to increase in the alerting condition (instead of being decreased as in the short SOA condition), compared to the no tone trials. In agreement with these assumptions, Asanowicz and Marzecová’s (2017) results showed that while the time of conflict processing increased in both short and long SOA conditions (compared to no alerting trials), in the former alerting also increased the error rates, while in the latter the error rates were decreased (see also Asanowicz et al., 2012) suggesting that accurate conflict resolution takes more time (in line with the idea of “deeper” processing by Craik and Lockhart, 1972). The question was therefore raised here whether the neural dynamics underlying conflict processing differed quantitatively and/or qualitatively in the two cases, or phases, of alerting.

## 2 Methods

### 2.1 Participants

The sample consisted of thirty-nine students (30 women) of the Jagiellonian University, who participated in the study in return for course credits. Four of them were rejected from EEG data analysis due to large EEG artifacts. The average age was 20.1 years (SD 1.4). None of the participants reported hearing impairments, all had normal or corrected-to-normal vision and no history of neurological disorders. Five participants reported left-handedness. Informed written consent was obtained from each participant before the experiment. The study was approved by the Ethics Committee of the Institute of Psychology at the Jagiellonian University.

### 2.2 Behavioral task: stimuli, procedure, and analysis

**Figure 1:**
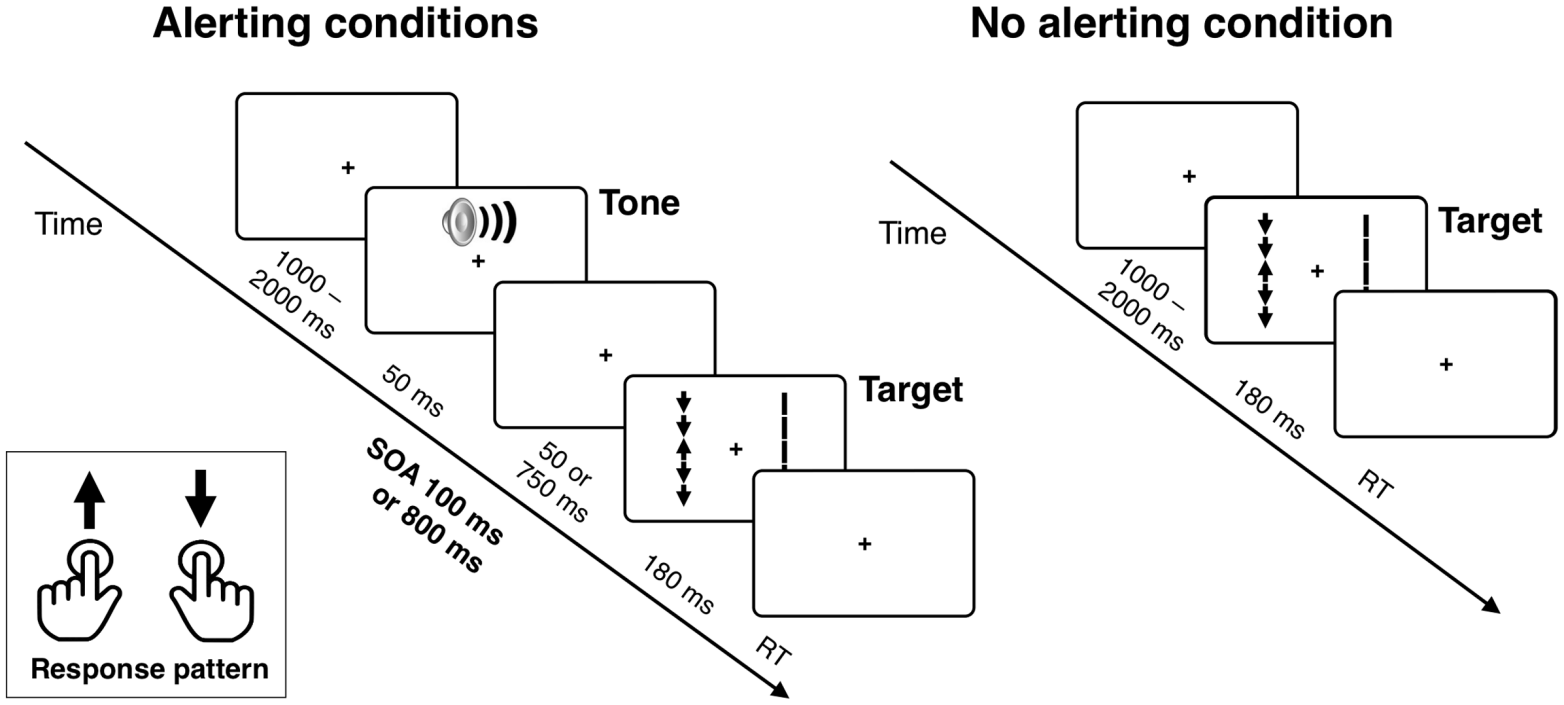
The stimuli used in the experimental task and sequence of events in a trial. The example shows the target arrow surrounded by incongruent flankers and the corresponding distractor set of five vertical lines (without arrowheads) simultaneously presented in the visual hemifield opposite to the target. In the alerting conditions, the target array is preceded by an alerting tone, presented with either 100 or 800 ms of stimulus onset asynchrony (SOA). See Methods for details.

The task is illustrated in Figure 1. Each trial of the task began with a fixation point displayed at the center of a computer screen. The time of the initial display of the fixation point varied randomly between 1,000 and 2,000 ms (in 150 ms steps, distributed uniformly). The fixation point was continuously displayed during the trial until response. Inter-trial interval was 500 ms. In two thirds of all trials, a 2,000 Hz, 50-ms alerting tone was presented before the target onset, with either 100 or 800-ms of stimulus onset asynchrony (SOA), constituting the SOA 100 and SOA 800 conditions (equinumerous). In the remaining one-third of trials the target was displayed immediately after the initial fixation (no tone condition).

The target stimulus was an arrow pointing either up or down, presented in the left or right visual field (similarly as in the Lateralized Attention Network Test or LANT in a study by Greene et al., 2008). The target was flanked by four additional arrows, two above and two below, that were either pointing in the same direction as the target arrow or the opposite direction (50/50) constituting the congruent and incongruent flanker conditions. A corresponding distractor stimulus set, consisting of five vertical lines without arrowheads, was simultaneously presented in the visual field opposite to the target (cf. Asanowicz et al., 2021; Evert et al., 2003). This bilateral stimulation enabled us to isolate a lateralized (contralateral vs. ipsilateral) EEG signal related to attentional selection of target stimuli from the overall visual activity (see below), and also diminished lateral exogenous orienting effects that could trigger horizontal eye movements. The bilateral arrays were presented for 180 ms, which is too short for volitional eye movements. All the variables were counterbalanced, whereas the condition order was randomized per participant.

The stimuli were presented at a viewing distance of roughly 56 cm. The fixation cross was 4 mm (0.4°). The target arrow and the flankers were each 8 mm (0.8°) long. The target and flanker arrowheads were 4 mm wide (0.4°). The length of all five (target and flanker) arrows in the display was 44 mm (4.4°). The arrows’ midpoints were displayed 18.5 mm (1.85°) to the left or right of the center of the screen. The distractor stimulus array consisted of five lines of the same length as the target and flanker arrows, but their width was increased by 1 mm to compensate for the absence of the arrow heads. All stimuli were black and were presented on a light gray background (RGB: 245, 245, 245). The stimuli were presented on a 21’ LCD monitor with a 60 Hz refresh rate. The ambient room lighting was approximately 30 lux, and the gray background on the monitor had a luminance of approximately 115 cd/m^2^. The tone was played through a set of standard computer stereo speakers, positioned close to the left and right sides of the display, at a comfortable volume level of approximately 45 dB SPL. PsychoPy software^1^ was used for experimental control.

Participants’ task was to identify the direction of the target (middle) arrow and respond by pressing the left or right Ctrl key on the computer keyboard using the left index finger for up-pointing targets and the right index finger for down-pointing targets. The response mapping was the same for all participants. Speed and accuracy of responses were measured. A new trial began automatically 500 ms after the response or after 2,000 ms if the participant did not respond. Participants were given written instructions and also received verbal instructions describing the task. They were explicitly asked to respond to target stimuli both as quickly and accurately as possible. Participants were not informed about the timing or frequency of the trials with a tone; they were only informed that the tone would be presented in some trials. We carefully instructed all participants to maintain central fixation, and explained why proper fixation was necessary during EEG measurements. The task began with two practice blocks consisting of 32 trials in total in which participants received accuracy feedback after each response. The practice session was followed by 672 experimental trials without feedback, divided into four blocks of 168 trials. The order of the trials was randomized within blocks individually for each participant. Between the blocks, participants were instructed to take breaks to rest their eyes. The task lasted about 1 h, and the whole session lasted for up to 120 min.

Before inferential statistical analysis of RT, trials with incorrect responses, or with RT faster than 200 ms or longer than 1,200 ms were excluded (in overall 4.3%, mean and SD of total RT (within-participant) remained unchanged after the trimming). The remaining correct response times, and percentages of errors were submitted into a 3 × 2 repeated-measures ANOVA with Tone (tone with 100 ms SOA, tone with 800 ms SOA, no tone) and Flanker (congruent, incongruent) as within-subject factors. The Greenhouse–Geisser correction was applied when the tone factor had three levels. Reported are corrected *df* and *p* values, and Greenhouse–Geisser *ε*. When the effect of tone was significant, 2 × 2 repeated-measures ANOVAs were performed for separate comparisons of the SOA 100 and SOA 800 conditions with the no tone condition.

### 2.3 EEG analyses

#### 2.3.1 EEG data recording and preprocessing

EEG was recorded using BioSemi ActiveTwo system with Ag-AgCl electrodes on 64 monopolar locations according to the extended 10–20 system, and two additional electrodes, the common mode sense (CMS) active electrode and the driven right leg (DRL) passive electrode, used as reference and ground electrodes, respectively.^2^ The vertical electrooculogram (EOG) was recorded from above and below the right eye, and horizontal EOG was recorded from the external canthi of both eyes. The data were recorded at a sampling rate of 1,024 Hz. Brain-Vision Analyzer software (version 2, Munich, Germany) was used for offline data preprocessing. Data were offline filtered with a 0.1–50 Hz band-pass and a 50 Hz band-rejection filter (Butterworth zero-phase FIR filters, attenuation of 12 dB/octave) and re-referenced to linked mastoids.

To maximize data quality, the following five steps of artifact correction and rejection were performed. First, to improve the detection of eye movements, gross artifacts were marked on any electrode with voltage differences greater than 500 μV within 2,500 ms intervals or voltage steps larger than 80 μV (the average amount of marked data per participant was below 1% of the continuous signal). Second, the ICA-based ocular correction (Jung et al., 2000) was applied to the continuous signal to remove eye movement artifacts. Third, the signal was split into segments from 1,200 ms before target onset to 1,500 ms afterward. Segmented data were baseline-corrected to a 200 ms epoch before the first stimulus onset. Segments with an incorrect response, or with RT longer than 1,200 ms were automatically excluded from further analyses. At the fourth step we addressed non-ocular artifacts, starting with the removal of segments with gross artifacts to optimize subsequent ICA-based artifact detection (criteria: overall minimum-maximum voltage differences > 500 μV or with voltage steps between adjacent data points > 80 μV; on average 2% of segments per participant were remove at this step, SD 5%, range 0–25%). Next, the remaining segments were corrected for residual non-ocular artifacts using the ICA (on average 1.5 components were removed per participant), and then checked again semi-automatically to reject segments with minimum-maximum voltage differences > 100 μV, voltage steps between adjacent data points > 50 μV, and absolute amplitudes > 150 μV. If necessary, the latter rejection criteria were adjusted according to signal characteristics in individual subjects (such as unusually generally small or large EEG amplitudes). On average 5% of segments per participants (SD 4%, range 0–11%) were excluded based on the latter criteria. Finally, in the fifth step, to exclude trials with horizontal eye movements toward the target, segments were marked when the horizontal EOG index (right minus left EOG) exceeded ±100 μV or voltage steps between adjacent data points exceeded 50 μV, within a time-window from −200 to 400 ms relative to target onset. On average 0.2% data per participant were removed based on these criteria (SD 0.3%, range 0–2%). The overall average number of accepted segments per participant was 592 (range 443–648). The average number of accepted segments per condition was 99 (range 75–109) for no-tone/congruent, 97 (72–110) for no-tone/incongruent, 100 (78–111) for SOA 100/congruent, 97 (70–111) for SOA 100/incongruent, 101 (71–111) for SOA 800/congruent, and 99 (74–111) for SOA 800/incongruent trials.

#### 2.3.2 Spatio-spectral source separation

Time-frequency analysis on the preprocessed data was conducted in MATLAB (MathWorks, v. 2023b) using custom-written scripts based on published scripts (Cohen, 2014b, 2017, 2022), and the Brainstorm toolbox (Tadel et al., 2011). For source separation, we used the generalized eigenvalue decomposition (GED), which is a feature-guided multivariate technique that effectively separates two covariance matrices of two *a priori*-specified features of interest (Cohen, 2022; de Cheveigné and Arzounian, 2015; Parra and Sajda, 2003). The first covariance matrix (*S*) is the channel (electrode) covariance of the relevant signal. The second matrix (R) is the channel covariance of the reference data. The GED can be generalized to the calculation of vectors that maximize the ratio of quadratic forms between symmetric matrices *S* and *R*. This principle can be expressed as: $R^{-1}\mathrm{SW}=\mathrm{\Lambda W}$ , where W represents the set of eigenvectors defining the spatial characteristics of the *S*/*R* power ratio, which in turn provides the parameters of the spatial component. i.e., the sensor weights constituting each spatial component. Each components’ importance is specified by their eigenvalues (Λ) that indicate the value of that *S*/*R* ratio.

Before computing the covariance matrices, the segmented and artifact-free single-trial data were filtered using the surface Laplacian. We used a 10th-order Legendre polynomial, and lambda was set at 1e–5, which effectively increases spatial selectivity and attenuates volume conduction confounds (Cohen, 2015). Separate GED analyzes were then conducted to derive spatial filters for the following defined a priori regions of interest (ROI): (1) the midfrontal (medial frontal) area, (2) the left and right centro-lateral (motor) areas, and (3) the left and right lateral occipital (visual) areas. Based on the assumption that spatially coherent neuronal populations generate oscillatory activity (e.g., Gross et al., 2001), each ROI’s spatial filter was constructed using the bandpass-filtered signal. This signal was obtained through filtering via the Hilbert method, using a Gaussian-shaped filter defined by the peak frequency and bandwidth with a full width at half maximum (FWHM). Both filter parameters were adjusted for each ROI, and each filter was applied to EEG data prior to calculation of the covariance matrices. The narrow-band covariance increased sensitivity of the GED for signals from locations of interest (i.e., brain areas with coherent oscillatory dynamics) and attenuate unwanted signals from other locations.

The covariance matrices were computed for each participant, separately for each task conditions and ROIs. Next, the obtained matrices were averaged across participants, which allowed for the extraction of the sources of EEG activity at the group level (cf. Van der Lubbe et al., 2025). A similar procedure has been used in previous group-level ICA analyses (Calhoun et al., 2001; Calhoun et al., 2009). Subsequently, the GED was applied to the group-averaged covariance matrices, which yielded a set of 64 spatial components (each being a weighted combination of all 64 channels), where component with the highest eigenvalue indicates the strongest contribution. For each ROI, a single component with the highest eigenvalue and best fit to the ROI’s spatial criteria (i.e., covering the specified brain areas) was retained for further analysis.

In the case of midfrontal area, our primary interest was in theta activity (cf. Asanowicz et al., 2022, 2023). Thus, the signal of matrix *S* was derived from the data filtered in using a Gaussian filter centered at 5 Hz with 4 Hz of FWHM. To maximize signal-to-noise in theta band, the signal of the reference matrix R was unfiltered. The parameters were based on prior studies localizing theta sources in the medial-frontal cortex (e.g., Scheeringa et al., 2008; Cohen and Donner, 2013; Asanowicz et al., 2022). Both matrices S and R were computed based on the 400–600 ms time window relative to target onset and contained the same trials. The first (largest) component showed the specified spatial pattern, maximized over the FCz electrode and it was selected for the medial-frontal source.

To isolate centro-lateral sources related to motor control of the responding hands, source separation was performed separately on trials with the right and left hand responses. To enhance the spatial precision of the resulting components, here we focused on motor-related ipsilateral alpha/mu activity, which indicates increased neural excitability over the motor cortex through contralateral desynchronization during response selection and execution (e.g., Van Wijk, 2022). To this end, the EEG data were filtered by a Gaussian filter centered at 12 Hz (FWHM = 5 Hz) for S and R matrices. For the left-hemisphere, both matrices were computed separately from trials with the left-hand response, and for the right-hemisphere—from trials with the right-hand response. To capture the response-related mu dynamics—i.e., desynchronization prior the execution and rebound afterward (e.g., Illman et al., 2022)—the time-windows for S and R matrices were defined relative to the time-point of response execution, from −200 to 0 ms and from 0 to 200 ms, respectively. Separation yielded the second highest and the highest eigenvalue for both motor components (left and right, respectively), along with a distinct spatial pattern reflecting motor-related activity.

To isolate the lateral occipital (visual) sources specifically related to processing information from the two relevant spatial locations (i.e., the locations in the left and right visual fields where the targets were presented), the source separation for the left and right hemispheres was performed separately on the data from trials with targets presented in the right and left visual fields, respectively. The S matrix derived from the signal filtered within alpha-band with peak of 10 Hz (FWHM = 2 Hz), and R matrix derived from broadband signal. Both covariances were calculated with a time-window spanning from 400 ms to 600 ms relative to target onset. The largest (first) component yielded from the left-target trials was selected as the left hemisphere visual source, and similarly, the largest component from the right-target trials was selected as the right visual source. In both cases, the topography of the component covered the lateral occipital areas.

To assess the anatomical distribution of the selected components, we employed a method involving the correlation of the forward models with the leadfield matrix. This matrix contains coefficients modeling the interaction between the source space and scalp sensors (Cohen and Gulbinaite, 2017; Hild and Nagarajan, 2009) and was generated using the Brainstorm toolbox’s Boundary Element Method (BEM) implementation. The forward models were constructed by multiplying the corresponding eigenvector with the covariance matrix S (Haufe et al., 2014). We mapped the resulting correlation coefficients onto the standard cortical surface (ICBM 125 MRI). Regions exceeding the 98th percentile threshold were highlighted in color (cf. Asanowicz et al., 2022; Van der Lubbe et al., 2023).

#### 2.3.3 Time-frequency decomposition of source-level activity

The Morlet wavelet transform was used to obtain the time-frequency representation of the signal from the separated sources. First, we reconstructed the time-series data for each source by multiplying the eigenvector of the selected component by the single-subject EEG signal (channels × time × trials). Then, the obtained source signal was convolved with 30 Gaussian-shaped versions of the mother wavelet, each with varying temporal resolution (Allen and MacKinnon, 2010), to decompose the time-series signal into time-frequency representation. The set of Morlet wavelets can be expressed as: $e^{i2\mathrm{\pi ft}}e^{-t^{2}/(2\sigma^{2})}$ , where i represents the complex operator, t is time, f denotes frequency ranging from 1 Hz to 30 Hz in 30 logarithmically spaced intervals, and *σ* is the width of the Gaussian related to each frequency band. The width was defined as σ=n/(2πf) , where n is the number of wavelet cycles. We adjusted the number of wavelet cycles from 3 to 8 in logarithmically spaced steps to achieve an optimal balance between temporal and frequency precision (Trujillo and Allen, 2007). The source’s signal reconstruction, subtractions, and then wavelet convolutions were done separately for each condition per participant. After the convolution, we extracted instantaneous EEG power and phase from the resulting complex signal. Specifically, we computed the squared magnitude to extract the EEG power and determined the phase angle at each time-frequency point. To obtain non-phase-locked (induced) power^3^, the phase-locked activity (i.e., the ERP, computed as the time-domain trial average) was subtracted from the time-domain single-trial EEG signal, before the described above time-frequency decomposition (cf. Asanowicz et al., 2021; Cohen and Donner, 2013). The obtained squared power values (μV^2^) were normalized as a percentage change relative to the pre-stimulus baseline at each frequency band: $\left[100\times ((W_{\left(f_{n},t\right)}-W_{(f_{n})|\text{baseline}})/W_{(f_{n})|\text{baseline}}))\right]$ . The baseline for the no- tone and SOA 100 conditions was computed using the time window from −500 to −300 ms relative to target onset, while for the SOA 800 condition—from −1,200 to −900 ms (as the tone was presented at −800 ms).

#### 2.3.4 Inter-source phase coherence (ISPC)

To assess source-level functional connectivity, we computed inter-site (here: source) phase coherence (ISPC) (Cohen et al., 2008; Lachaux et al., 1999) between pairs of obtained GED components (sources). To this end, we used the instantaneous phase angles at each time- frequency point from the wavelet convolution of Laplacian-transformed and the GED-reconstructed signal (as described above). Inter-areal phase synchronizations in the EEG signal are thought to capture periodic interactions, generated by the mechanism of spike-timing coordination among neural assemblies (Fries, 2005; Siegel et al., 2012). ISPC is defined as complex phase angle differences between sites across trials, according to the formula:


$$\mathrm{ISP}C_{\left(f_{n},t\right)}=|\frac{1}{k}\sum_{t=1}^{k}e^{i(\Phi_{x}(f_{n},t)-\Phi_{y}(f_{n},t)).}|$$

where, x and y represent two distinct sources, and Φ denotes the phase value of single sample ( $f_{n},t)$ . The resulting ISPC index varies between 0 and 1, where 0 indicates no phase synchrony between two sites (i.e., random distributed phases), while 1 indicates perfect phase synchrony between two sites.

#### 2.3.5 Measurement and analysis of EEG power and ISPC

To examine target location-related and responding hand-related ipsilateral vs. contralateral modulations of local power and ISPC, we calculated lateralized power spectra (LPS)—for power, and lateralized phase coherence spectra (LP_C_S)—for ISPC, based on the method described by Van der Lubbe and Utzerath (2013) (see alsoVan der Lubbe et al., 2023). The LPS indices were calculated by a double subtraction of raw (i.e., not baseline-normalized) time-frequency data from the left and right sources at each time-frequency point. First, the ipsilateral–contralateral subtraction was calculated separately for segments with targets in the left and right visual fields, and for segments with the left- and right-hand responses, then scaled by the sum of activation from both hemispheres (ipsilateral + contralateral), and averaged, according to the formula:


$$\mathrm{LP}S_{\left(f_{n},t\right)}=\frac{W_{(f_{n},t)|\text{ipsi}}-W_{(f_{n},t)|\text{contra}}}{W_{(f_{n},t)|\text{ipsi}}+W_{(f_{n},t)|\text{contra}}}$$

where $\;W_{\text{ipsi}}$ is trial-averaged signal from the ipsilateral source (relative to target visual field), $\;W_{\text{contra}}$ is signal from the contralateral source; both measured within the same frequency- band ( $f_{n}$ ) and time-window. The LPS values vary from −1 to +1. A positive LPS value indicates larger power or ISPC at the ipsilateral site relative to the contralateral site, and zero indicates no hemispherical difference.

To select time-frequency data for the statistical analyses, we used a two-step data-driven strategy, intended to improve measurement sensitivity while avoiding circular inferences (cf. e.g., Cohen, 2014b). First, frequency windows for EEG power and ISPC measurements were selected on condition-averaged time-frequency plots. We selected the frequency ranges that exhibited the most robust activity within 0–1,000 ms relative to target onset (and from −500 to 500 ms relative to response onset in the response-locked data), independently of any specific prior predictions. Second, time windows for power and ISPC analyses were selected on the condition-averaged waveforms (presented in the time-domain) of the frequency-band selected in the first step. At this step, we used the methods of collapsed localizer and functional localizer (Luck and Gaspelin, 2017). The collapsed localizer was used for midfrontal power, in which the time-window is selected simply on condition-averaged data. The functional localizer was used for measurements of lateralized activity related to visual- and motor processing, i.e., from the lateral parieto-occipital and lateral centro-parietal sources. Functional localizer is based on a hypothesis-driven contrast; here, it is the hypothesis determining the specific contralateral vs. ipsilateral hemispheric differences relative to the cued location and responding hand, respectively. Thus, the time-windows were determined on the task- and condition-averaged contra-ipsilateral differences, assumed to represent specifically the attention- and motor-related activity. In both localizer methods, we measured mean activity beginning at the time-point at which the increasing waveform of interest reached 50% of its total amplitude, and ending at the time point at which the falling waveform of interest reached the same 50% value of its amplitude. The search window was again from 0 to 1,000 ms relative to target onset (and from −500 to 500 ms relative to response onset in the response-locked data).

Local power from the midfrontal source was measured in stimulus-locked data within the 4–6 Hz frequency range, from 270 to 850 ms after target onset. Local power from the centro-lateral (motor) sources was analyzed in the stimulus-and response-locked data. Stimulus-locked LPS was measured in time-frequency windows of 4–8 Hz and 410–870 ms for theta power, 10–13 Hz and 450–1,230 ms for alpha/mu power, and 18–23 Hz and 470–900 ms for beta power. Response-locked LPS was measured in windows of 4–8 Hz, from −140 to 150 ms for theta power, 10–13 Hz from −170 to 250 ms for alpha/mu power, and 18–23 Hz from –190 to 250 ms for beta power. LPS of local power from the occipital (visual) sources was measured in stimulus-locked data within a window of 10–13 Hz from 270 to 850 ms after target onset. For connectivity analysis, stimulus-locked LP_C_S of ISPC between the midfrontal and lateral occipital (visual) sources was measured in frequency-windows of high theta (6–8 Hz) from 160 to 360 relative to target onset, and alpha (8–12 Hz) from 500 to 860 ms relative to target onset. Stimulus-locked locked LP_C_S of ISPC between the midfrontal and motor sources was measured in windows of 3–8 Hz from 460 to 900 ms, and 8–13 Hz from 490 to 620 ms relative to target onset. Lastly, response-locked LP_C_S of ISPC between the centro-lateral (motor) and lateral occipital (visual) was measured in in a window of 3–7 Hz from −150 to 220 ms relative to response execution.

#### 2.3.6 ERP measurement and analysis

Before ERP analysis, artifact-free segments were averaged over each condition separately for each participant. To select the ERPs for the statistical analyses, we used the same localizer methods as for the time-frequency data, except the step of frequency selection.

To measure the target-evoked midfrontal N2, we used a baseline-independent peak-to-peak method because the tone presentations affected the midfrontal negativity so that voltage differed between tone conditions already before target onset (cf. Asanowicz et al., 2022; Böckler et al., 2011). The N2 amplitudes were measured at FCz as mean amplitude 260–340 ms after target onset relative to the preceding P2 measured as mean amplitude 210–240 ms after target onset.

To measure the posterior contralateral negativity (PCN) in the ERP, we calculated event-related lateralizations or ERLs (Wascher and Wauschkuhn, 1996) from activity recorded over visual cortex at PO7 and PO8, as the average of contra–ipsilateral differences for trials with the LVF and RVF targets, by the formula: ((PO8_LVF_ − PO7_LVF_) + (PO7_RVF_ – PO8_RVF_)) / 2. Thus, a negative ERL value indicates larger negativity at the hemisphere contralateral to the target visual field. The N2pc component of the PCN was measured as mean amplitude 260–340 ms after target onset, and the sustained posterior contralateral negativity (SPCN) as mean amplitude 320–580 ms. The latencies of the PCN components did not differ between the conditions, thus, their analysis is not reported. To obtain topographies of the ERLs, we subtracted all symmetrical electrodes and plotted the averaged contra–ipsilateral differences on the left hemisphere.

To measure the LRP, we calculated ERLs from activity recorded over motor cortex at the C3 and C4 sites, as the average of the difference contra–ipsilateral relative to the responding hand: ((C4_Left Hand_ − C3_Left Hand_) + (C3_Right Hand_ − C4_Right Hand_)) / 2 (Coles, 1989), so that a negative value reflects activation of the correct response. In the stimulus-locked averages, the stimulus-locked LRP amplitudes were measured as mean amplitudes 260–560 ms relative to target onset, and response-locked LRP as mean amplitudes from −170 to –40 ms relative to the response execution time point. The latency of the stimulus-locked LRP was evaluated using the JackKnife method (Kiesel et al., 2008; Ulrich and Miller, 2001). Peak latencies were measured in one-leave-out grand means, in the time window of 0–700 ms in the stimulus-locked data (and from −400 to 0 ms in the response-locked data) on waveforms low-pass filtered at 20 Hz (this filter was not applied for the amplitude measurements). The diminished error variance was corrected by dividing *F* values by (N – 1)^2^. Additionally, we measured LRP onset latencies using the JackKnife averaging and 20% fractional peak latency (cf. Luck, 2014). The latency of response-locked LRP was not tested because no differences were present between the averages of the experimental conditions.

#### 2.3.7 Inferential statistics

For inferential statistics, EEG power, ISPC, and ERP data were submitted into a 2 × 3 repeated-measures ANOVA with and Flanker (congruent, incongruent) and Tone (no-tone, SOA 100, SOA 800) as within-subject factors. The Greenhouse–Geisser correction was applied when the Tone factor had more than two levels (i.e., more than one degree of freedom in the numerator) to adjust for violations of sphericity. Reported are corrected *df* and *p* values, and Greenhouse–Geisser *ε*. When the main effect of Tone or the Tone × Flanker interaction were significant, further ANOVAs were carried out separately for the SOA 100 and SOA 800 (in both cases with comparison to the no tone condition) to identify the possible differences between phasic and tonic alerting (cf. e.g., Asanowicz and Marzecová, 2017). Additionally, the ERL, LPS, and LP_C_S indices were tested against zero using one-sample *t* test to assess the statistical significance of the ipsilateral vs. contralateral differences (all reported *p* values are two-sided).

## 3 Results

### 3.1 Behavioral results

**Figure 2:**
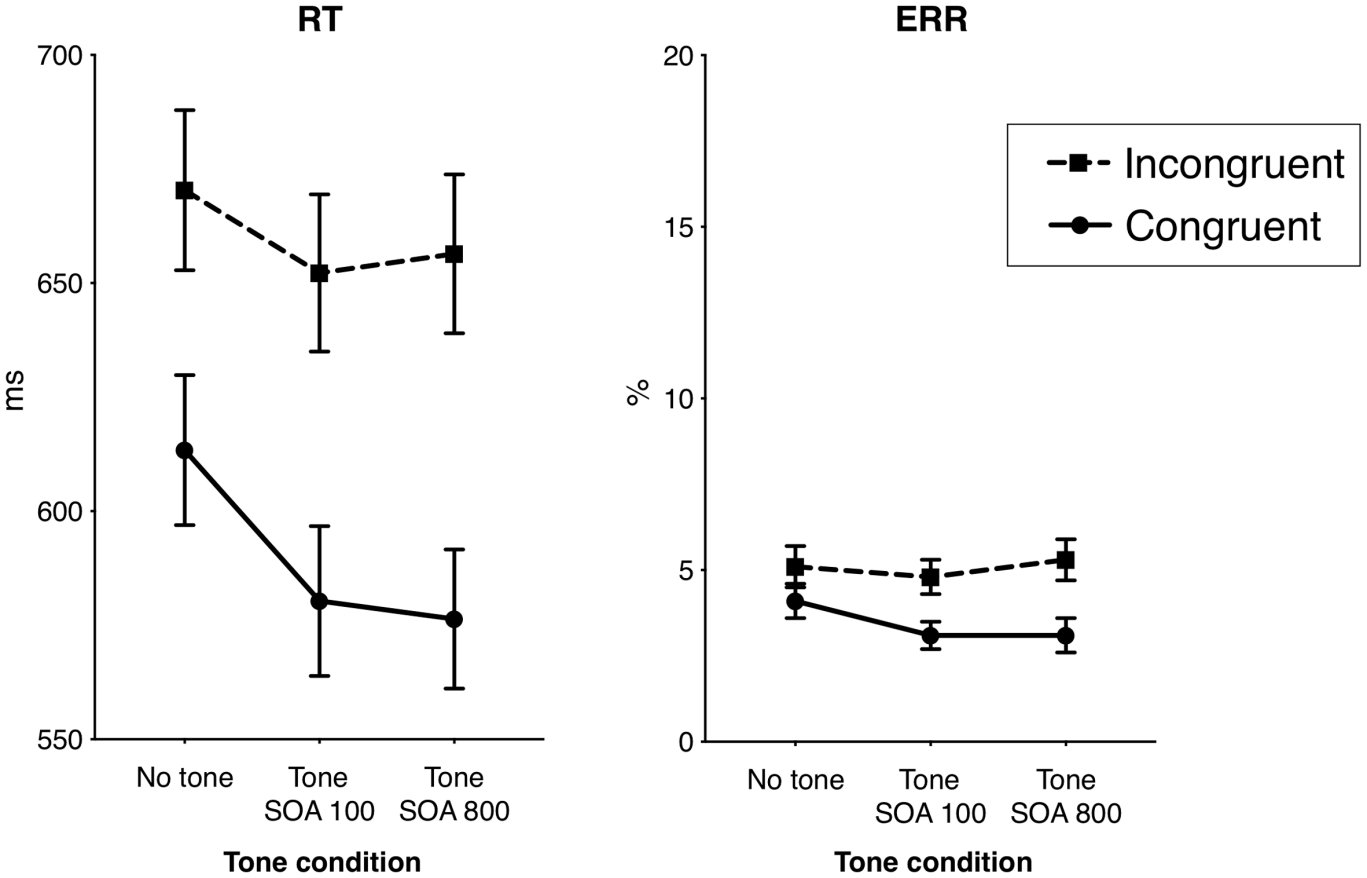
Behavioral results: the effects of flanker congruency and alerting tone on the response times (RT) of correct responses and the error rates (ERR). Vertical bars represent standard errors of the mean.

Mean response times (RT) and error rates (ERR) for each task condition are shown in Table 1 and Figure 2. The overall mean time of correct responses was 624 ms (SD 102). The overall mean ERR was 4.2% (SD 2%). The main effect of Tone was significant in RT, *F*_1.55,58.80_ = 45.41, *p* < 0.001, *η_p_^2^* = 0.54, *ε* = 0.77, but not in ERR, *F*_1.80,68.53_ = 1.47, *p* = 0.24, *η_p_^2^* = 0.04, *ε* = 0.90. Specifically, the overall RT in the no tone condition (642 ms) was significantly longer compared to the SOA 100 condition (616 ms), *F*_1,38_ = 113.02, *p* < 0.001, *η_p_^2^* = 0.75, and to the SOA 800 condition (also 616 ms) *F*_1,38_ = 44.62, *p* < 0.001, *η_p_^2^* = 0.54. (The difference between the overall RT in the two SOA conditions was not significant, *F* < 1.0.) Further, responses were generally faster in the congruent flanker condition than in the incongruent condition: 590 vs. 660 ms, *F*_1,38_ = 117.87, *p* < 0.001, *η_p_^2^* = 0.76, as well as less error prone: 3 vs. 5%, *F*_1,38_ = 17.26, *p* < 0.001, *η_p_^2^* = 0.31, producing the typical flanker effect.

**Table 1:** Average response time (RT) of correct responses and average error rate (ERR), with 95% confidence Intervals (CI), for each experimental condition.

Alerting condition	Flanker condition	RT		ERR	
		Mean (SD)	95% CI	Mean (SD)	95% CI
No tone	Congruent	613 (103)	580–647	4.1 (3.1)	3.1–5.1
	Incongruent	670 (110)	635–706	5.1 (3.6)	3.9–6.3
SOA 100	Congruent	580 (102)	547–614	3.1 (2.6)	2.3–4
	Incongruent	652 (108)	617–687	4.8 (3.4)	3.7–5.9
SOA 800	Congruent	576 (95)	546–607	3.1 (2.8)	2.2–4
	Incongruent	656 (109)	621–692	5.3 (3.9)	4–6.5

The Flanker × Tone interaction was significant in RT, *F*_1.77,67.26_ = 23.82, *p* < 0.001, *η_p_^2^* = 0.39, *ε* = 0.89, and not in ERR, *F*_1.98,75.36_ = 2.93, *p* = 0.15, *η_p_^2^* = 0.05, *ε* = 0.99. Analyzed separately, the Flanker × Tone interaction in RT was significant both in the SOA 100, *F*_1,38_ = 15.85, *p* < 0.001, *η_p_^2^* = 0.29, and in the SOA 800 condition, *F*_1,38_ = 72.00, *p* < 0.001, *η_p_^2^* = 0.66. The interactions indicated that the flanker effect (incongruent *minus* congruent) was smaller in the no tone trials (57 ms) than in the trials with the tone (SOA 100: 72 ms, and SOA 800: 80 ms). Also the difference between the flanker effect in the SOA 100 and SOA 800 was significant, *F*_1,38_ = 5.09, *p* = 0.030, *η_p_^2^* = 0.12, indicating a larger flanker effect in the SOA 800.

### 3.2 GED components

**Figure 3:**
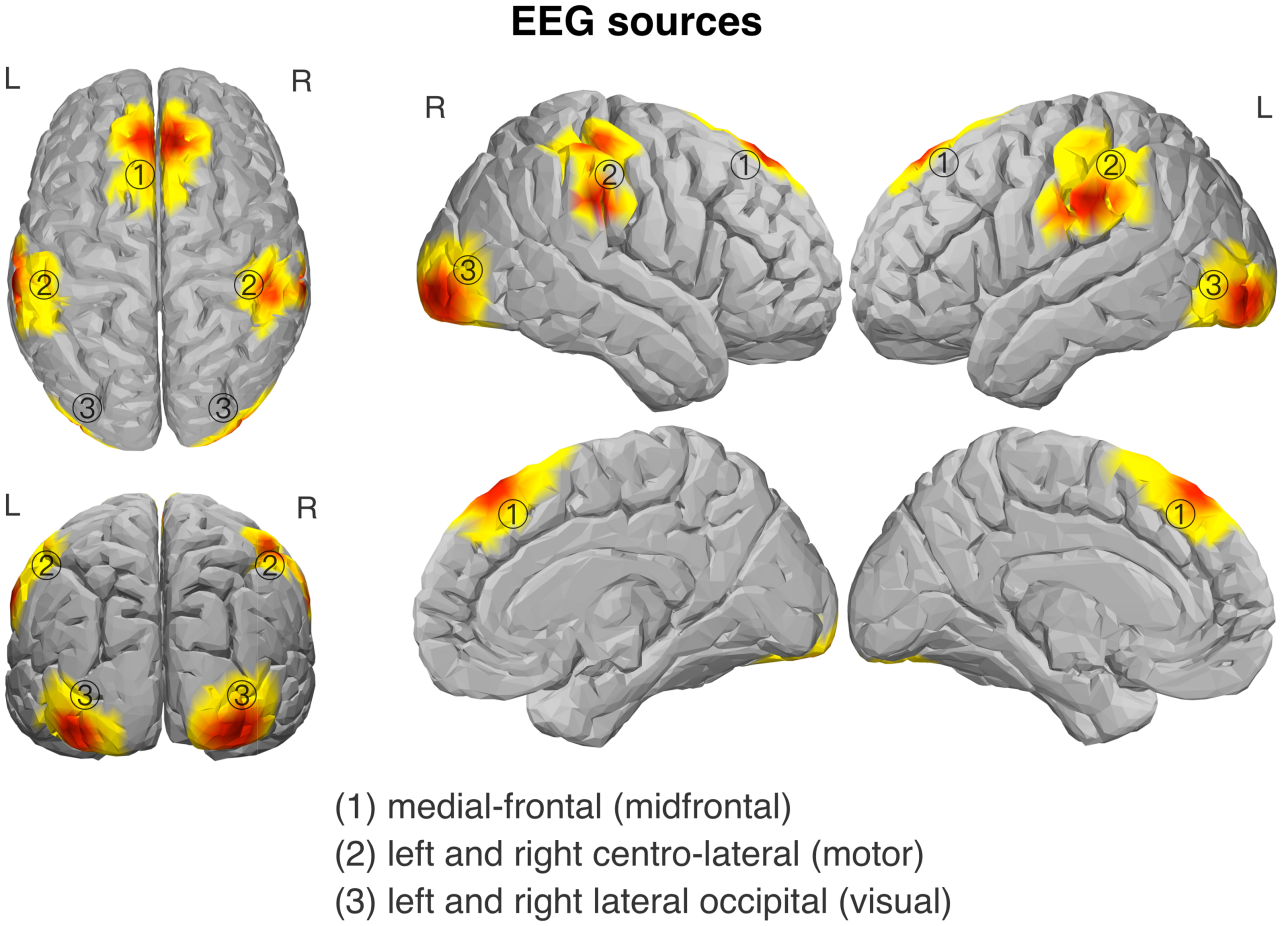
Topography of the isolated source components of EEG signal: (1) midfrontal source; (2) centro-lateral (motor) sources, related to response processing (obtained separately for left- and right-hand trials); and (3) lateral occipital (visual) sources, related to visual target processing (obtained separately for the left and right target stimuli).

By means of the GED-based method of EEG source separation we isolated the three a-priori specified sources: (1) midfrontal, (2) left and right centro-lateral (motor), and (3) left and right lateral occipital (visual). Visualizations of the spatial distributions of the isolated sources are shown in Figure 3. The subsequently estimated source-level time-frequency activity was used to examine local power within the sources and phase coherence-based functional connectivity between the sources.

### 3.3 Midfrontal activity

#### 3.3.1 Midfrontal theta power

**Figure 4:**
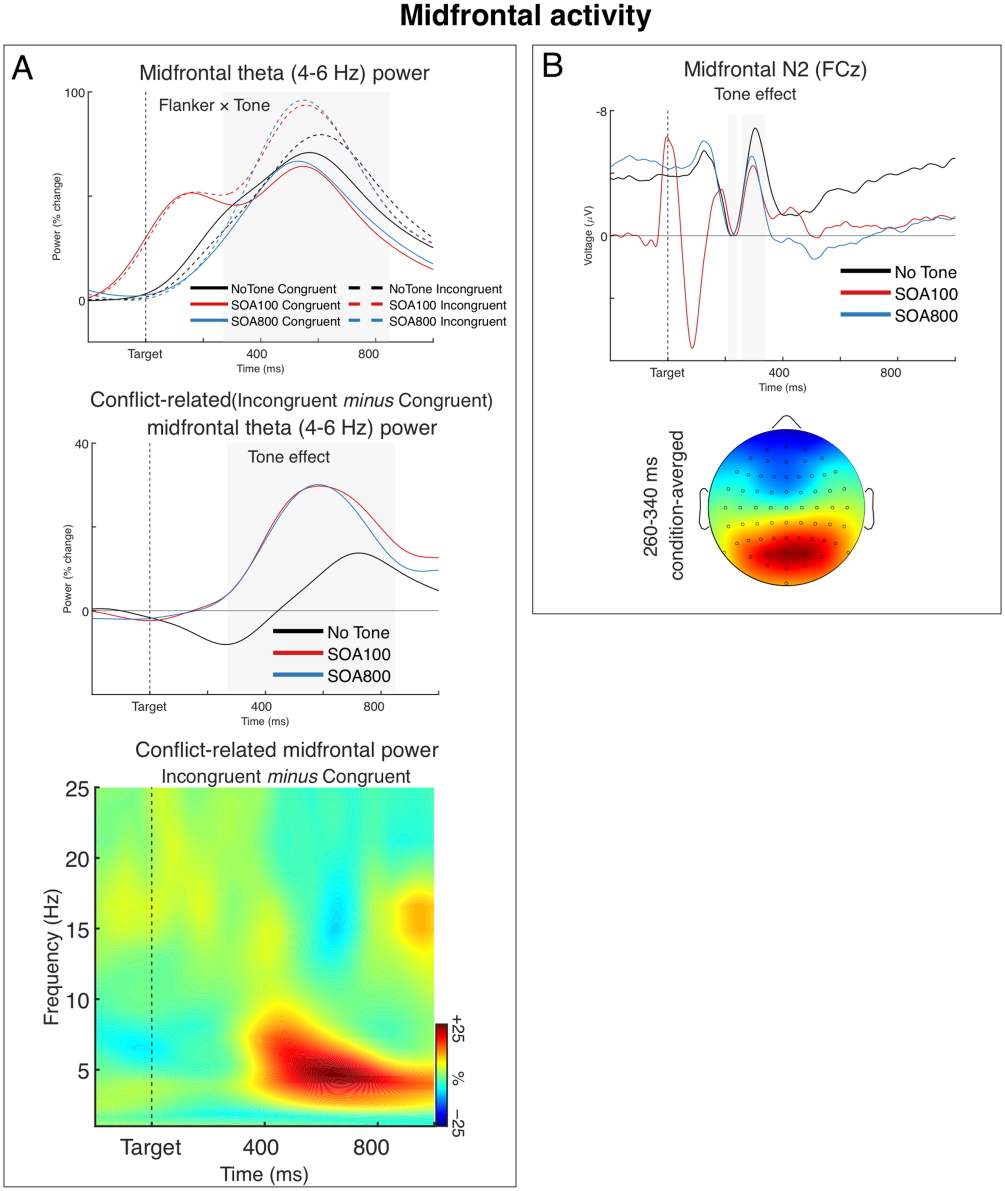
Midfrontal (medial frontal) activity. **(A)** Post-target modulations of induced (non-phase-locked) theta power from the midfrontal source. The upper chart shows grand averages of theta power over time for each task condition. The chart shows that alerting tone modulated theta power predominantly in the incongruent flanker condition. Note that the early peak in the SOA 100 condition (red lines) is an effect of the tone presented 100 ms before target onset. The grey-shaded area marks the analyzed time window. The middle chart shows grand averages of the incongruent–congruent differences in midfrontal theta power, illustrating the alerting effect on the conflict-related theta. The time-frequency plot at the bottom shows the spectra of the incongruent-congruent difference in midfrontal power (i.e., conflict-related theta) averaged across the three Tone conditions. **(B)** The Tone effect on the N2 component of the ERP and condition-averaged topography of the N2. Negative voltage points upwards. The N2 amplitude was measured relative to the preceding P2 (instead of the prestimulus baseline, which was affected by Tone condition). To show the N2-to-P2 amplitude differences, we present the waveforms aligned (baselined) to the P2 epoch, so that the mean P2 amplitudes (210–240 ms) are at zero and the negative values of the N2 peaks show the actual N2–P2 amplitude differences. The gray areas indicate the time windows within the P2 and N2 mean amplitudes were measured. The head map is min–max scaled, with positive polarity in red, negative polarity in blue.

Figure 4A shows grand averages of theta power from the midfrontal source for each of the Flanker and Tone combination (upper panel), averages of the conflict-related theta (i.e., incongruent–congruent difference) for the three Tone conditions (middle panel), and a time-frequency plot of the conflict-related power modulation from midfrontal source averaged across the three Tone conditions. The results showed that flanker incongruence entailed the typical increase of midfrontal theta power, compared to the congruent flanker condition (cf. Cavanagh and Frank, 2014; Cavanagh and Cohen, 2022). Importantly, this conflict-related increase of theta power was larger in the two alerting conditions (SOA 100 and 800) than in the no-tone condition. Specifically, as seen in Figure 4A (upper panel), midfrontal theta power increased in the incongruent trials of the two alerting conditions about twice as much as in the no-tone condition (relative to the congruent condition). Importantly, the alerting affected mostly the incongruent trials; in the congruent trials, theta power remained similar regardless of the alerting conditions.

The ANOVA confirmed these observations. The main effect of Flanker was significant, *F*_1,34_ = 12.49, *p* < 0.001, *η_p_^2^* = 0.27, and so was the 2 × 3 interaction between Flanker and Tone, *F*_1.91,65.06_ = 3.42, *p* = 0.041, *η_p_^2^* = 0.09, *ε* = 0.96, while the main effect of Tone was not significant, *F* < 1.0. When we tested alerting effects separately for the two SOAs, the 2 × 2 interaction between Flanker and Tone (no tone, tone with SOA 100 or SOA 800) was significant both for SOA 100 (Flanker × Tone: *F*_1,34_ = 4.79, *p* = 0.036, *η_p_^2^* = 0.12, main effect of flanker: *F*_1,34_ = 6.34, *p* = 0.017, *η_p_^2^* = 0.16) and SOA 800 (Tone × Flanker: *F*_1,34_ = 4.48, *p* = 0.042, *η_p_^2^* = 0.12, main effect of flanker: *F*_1,34_ = 7.57, *p* = 0.009, *η_p_^2^* = 0.18). Lastly, there was no significant difference between the two SOA conditions (Flanker × Tone [SOA 100, SOA 800]: *F* < 1.0, n.s.).

#### 3.3.2 Midfrontal N2

The midfrontal N2 component of the ERP is shown in Figure 4B. As seen in the figure, the N2 reached its maximum about 310 ms after target onset. Interestingly, while the results showed a clear alerting effect on the N2 (see Figure 4B), there was neither a difference between the flanker conditions nor a Flanker × Tone interaction. Nevertheless, the lack of flanker N2 effect is not a surprise as it has been already reported in several publications (Asanowicz et al., 2019; Kałamała et al., 2018; Tillman and Wiens, 2011).

The ANOVA confirmed the main effect of Tone, *F*_1.95,66.31_ = 6.81, *p* = 0.002, *η_p_^2^* = 0.17, *ε* = 0.98, while the main effect of Flanker and the Flanker × Tone interaction were not significant, *F*s < 1.0, n.s. Sperate 2 × 2 ANOVAs showed that the N2 amplitude in no-tone condition (no alerting) was significantly larger than in both SOA 100, *F*_1,34_ = 10.82, *p* = 0.002, *η_p_^2^* = 0.24, and SOA 800 conditions, *F*_1,34_ = 8.51, *p* = 0.006, *η_p_^2^* = 0.20, while there was no significant difference between the two alerting conditions (SOA 100 and 800), *F* < 1.0, n.s.

### 3.4 Motor activity

In this section we describe the effects of Tone and Flanker manipulations on the lateralized motor activity measured as non-phase-locked LPS and the LRP component of the ERP. Both measures were analyzed in stimulus-locked and response-locked data segmentations. However, we did not find any significant effects of alerting in the latter, thus we do not report these results.

#### 3.4.1 Response selection-related power

**Figure 5:**
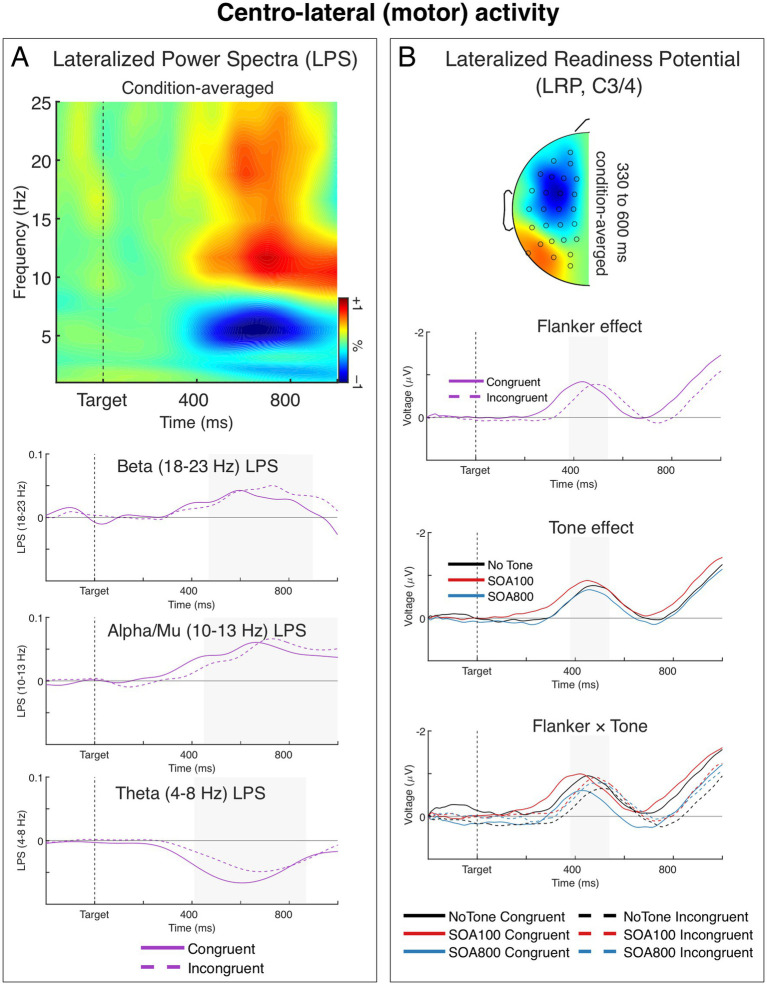
Response-related centro-lateral activity. **(A)** Lateralized power spectra (LPS) (relative to the responding hand) of stimulus-locked induced power from the centro-lateral (motor) sources. The time-frequency plot shows condition-averaged LPS from the motor sources. The LPS results show a significant transient lateralization of power in the beta (∼18–23 Hz), alpha/mu (∼10–13 Hz), and theta (∼4–8 Hz) bands, indicating that contralateral beta and mu power was smaller than ipsilateral power, and contralateral theta power was larger than ipsilateral theta power. The charts below show LPS in the beta, alpha/mu, and theta bands over time for the two flanker conditions. Tone effects are not shown in the figure, as no significant effects of tone were observed. **(B)** Stimulus-locked lateralized readiness potential (LRP) recorded over the motor cortex (the C3/4 sites). The head map show topography of condition-averaged LRP. The topographic map was obtained by subtracting all the symmetrical electrodes (contra–ipsilateral) and plotted the difference on the left hemisphere. The map is min–max scaled, with positive polarity in red and negative polarity in blue. The head view is from above. The charts below show the LRPs separately as a function of flanker congruency, alerting tone, and Flanker × Tone interaction. All three effects were significant in the stimulus-locked LRPs. Negative voltage (plotted upward) represents activation of the correct response (contralateral to the responding hand).

A time-frequency plot of stimulus-locked LPS from the motor sources averaged across Flanker and Tone conditions is shown in Figure 5A. As seen in the figure, the response-related lateralization was present in the beta, alpha/mu, and theta bands. The beta and mu lateralizations were present as a positive LPS deflection, indicating a stronger contralateral vs. ipsilateral decrease of beta and alpha/mu power (relative to responding hand). The theta lateralization was present as a negative LPS deflection indicating a stronger contralateral vs. ipsilateral increase of theta power (relative to responding hand). These findings are in line with previous studies (see, e.g., Asanowicz et al., 2021; Van der Lubbe et al., 2025). We therefore analyzed LPS in all the three bands. Grand averages of the LPS in the beta, mu, and theta bands for two flanker conditions (averaged across Tone conditions) are shown in Figure 5A.

The beta power (18–23 Hz) contralateral reduction was significantly stronger in the incongruent than in congruent condition, *F*_1,34_ = 4.15, *p* = 0.049, *η_p_^2^* = 0.11 (cf. e.g., Asanowicz et al., 2021; Van der Lubbe et al., 2025). This difference was mainly due to a lengthening of the beta lateralization in the incongruent flanker condition (see Figure 5A). None of Tone effects were significant, *F* < 1.0. The beta LPS was significantly larger than zero (indicating the ipsilateral–contralateral difference) in both congruent and incongruent flanker conditions, *t*_34_ = 5.36 and 6.78, respectively, *p*’s < 0.001.

The contralateral reduction of mu power (10–13 Hz) was also stronger in the incongruent than in congruent condition, *F*_1,34_ = 4.55, *p* = 0.040, *η_p_^2^* = 0.12. Moreover, here we found a significant 2 × 3 Flanker by Tone interaction, *F*_1.85,62.81_ = 4.77, *p* = 0.014, *η_p_^2^* = 0.12, *ε* = 0.92. When we tested alerting effects separately for the two SOAs, the 2 × 2 interaction between Flanker and Tone (no tone, tone with SOA 100 or SOA 800) was not significant for SOA 100 (Flanker × Tone: *F* < 1.0, n.s., main effect of flanker: *F*_1,34_ = 12.51, *p* = 0.001, *η_p_^2^* = 0.27), whereas it was significant for SOA 800 (Tone × Flanker: *F*_1,34_ = 8.24, *p* = 0.007, *η_p_^2^* = 0.20, Flanker effect in SOA 800: *F* < 1.0). In other words, the tone presented 800 ms before target onset eliminated the flanker effect on the response-related lateralization of local mu power (which was otherwise present both in no-tone and SOA 100 conditions). All the six analyzed here mu LPS indices were significantly larger than zero (indicating the ipsilateral vs. contralateral difference), *t*_34_ ≥ 3.47, *p* ≤ 0.001.

Lastly, the contralateral increase of theta power (4–8 Hz) (indicated by a negative LPS, see Figure 5A) was significantly larger in the congruent than incongruent condition, *F*_1,34_ = 5.70, *p* = 0.023, *η_p_^2^* = 0.14 (cf. e.g., Asanowicz et al., 2021; Van der Lubbe et al., 2025). The Tone effects were not significant, *F* ≤ 1.6, *p* ≥ 0.20, *η_p_^2^* ≤ 0.0.4. Theta LPS was significantly larger than zero (indicating the ipsilateral vs. contralateral difference) in both flanker conditions, *t*_34_ ≥ 4.68, *p* < 0.001.

#### 3.4.2 Response selection-related ERPs: LRPs

Figure 5B shows grand averages and topographies of the stimulus-locked LRPs from the sites C3/4 located over the motor cortex. In the congruent trials, the LRP diverged from zero at about 314 ms after target onset and formed a negative wave reflecting activation of the correct response. In the incongruent trials, the LRP emerged about 70 ms later, which corresponds with the 70 ms flanker effect in RT. Analysis of the LRP peak latencies confirmed the Flanker main effect, *F*_1,34_ = 71.95, *p* < 0.001, *η_p_^2^* = 0.68. The ANOVA also showed a significant main effect of Tone, *F*_2,68_ = 84.81, *p* < 0.001, *η_p_^2^* = 0.71. Examining the main effect of Tone we found that, while the LPR peak latency was generally shorter in the tone trials than in the no-tone trials, the Tone effect was slightly larger for SOA 100 (412 vs. 498 ms, *F*_1,34_ = 166.69, *p* < 0.001, *η_p_^2^* = 0.83) than for SOA 800 (440 vs. 498 ms, *F*_1,34_ = 59.15, *p* < 0.001, *η_p_^2^* = 0.64), indicating that the alerting tone shortened the LRP peak latency more in the SOA 100 than in SOA 800 condition.

Moreover, also the Flanker × Tone interaction was significant, *F*_2,68_ = 4.63, *p* = 0.013, *η_p_^2^* = 0.12. Examining this 2 × 3 interaction we found that in the no-tone vs. SOA 100 comparison, the Flanker × Tone interaction was not significant, *F* < 1.0. Whereas in the no-tone vs. SOA 800 comparison, the Flanker × Tone interaction was significant *F*_1,34_ = 6.02, *p* = 0.019, *η_p_^2^* = 0.15, indicating that the flanker effect (incongruent–congruent) on the LRP peak latencies was slightly larger in SOA 800 condition than in the no-tone condition (77 vs. 45 ms, respectively, see Figure 5B). The larger flanker effect in SOA 800 was further confirmed when it was with SOA 100: here the Flanker × Tone interaction was also significant, *F*_1,34_ = 9.56, *p* = 0.004, *η_p_^2^* = 0.22, reflecting a larger flanker effect in SOA 800 than in SOA 100 (77 vs. 46 ms).

Additionally, we measured LRP onset latencies to examine whether the observed alerting effects on the LRP peak latency were not due to an overall waveform time shift. The results showed only the flanker main effect, *F*_1,34_ = 10.29, *p* = 0.003, *η_p_^2^* = 0.22 (other effects: *F*s < 1.0, n.s.).

### 3.5 Visual activity

Introducing bilateral stimulation to the flanker task and applying the double subtraction method enabled us to isolate the stimulus selection-related lateralized signal from the overall visual activity (cf. Asanowicz et al., 2021). The effects of flanker interference on visual selection are indicated here by lateralized alpha power, and the N2pc and SPCN components of the ERP.

#### 3.5.1 Stimulus selection-related alpha power

**Figure 6:**
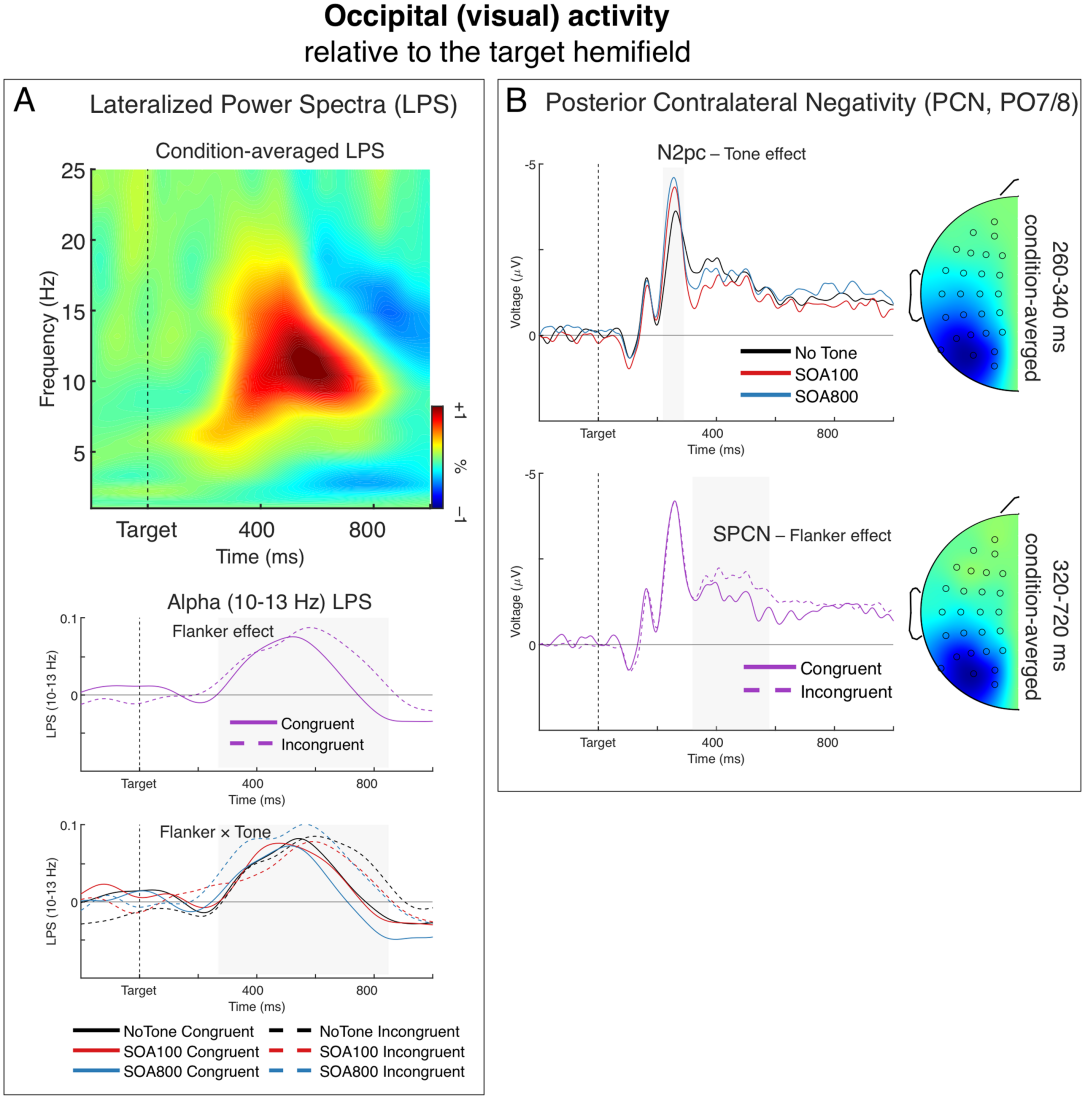
Lateralized occipital (visual) activity, relative to the target visual hemifield. **(A)** Lateralized power spectra (LPS) of stimulus-locked induced power from the occipital sources. The time-frequency plot depicts condition-averaged LPS. The LPS result shows a significant transient lateralization of power in the alpha-band (∼10–13 Hz) indicating that contralateral alpha power was smaller than ipsilateral alpha power. The charts below show this alpha-band LPS over time separately for the main effect of Flanker (middle) and the Flanker × Tone interaction (bottom). **(B)** The posterior contralateral negativity (PCN) of the ERP. Negative voltage is plotted upward. The upper chart depicts the effect of Tone on the N2pc component (marked by the grey-shaded area). The head map shows topography of condition-averaged N2pc. The bottom chart depicts the effect of Flanker on the SPCN component (marked by the grey-shaded area). The head map shows topography of condition-averaged SPCN. The topographic maps were obtained by subtracting all the symmetrical electrodes (contra–ipsilateral) and plotted the difference on the left hemisphere. The maps are min–max scaled, with positive polarity in red and negative polarity in blue. The head view is from above.

Figure 6A shows a time-frequency representation of LPS from the occipital (visual) sources averaged across Flanker and Tone conditions (upper panel), grand averages of LPS in the alpha band (10–13 Hz) for the two Flanker conditions illustrating the main effect of flanker congruence (middle panel), and grand averages of alpha-band LPS illustrating the Flanker by Tone interaction (lower panel). Alpha-band LPS formed a positive deflection indicating a contralateral alpha power decrease, which reached maximum between 500 and 700 ms after target onset. This alpha LPS was significantly larger than zero in each of the six Flanker × Tone conditions, *t*_34_ ≥ 2.70, *p* ≤ 0.011 (indicating significant ipsilateral vs. contralateral differences).

The alpha power reduction, as indicated by the positive LPS deflection, was significantly larger in the incongruent trials than in congruent trials, *F*_1,34_ = 11.11, *p* = 0.002, *η_p_^2^* = 0.25 (see the middle panel of Figure 6A), which replicates our previous findings (Asanowicz et al., 2021, 2022). The main effect of Tone was not significant, *F* < 1.0, but the Flanker × Tone interaction reached the significance level, *F*_1.97,66.88_ = 3.42, *p* = 0.039, *η_p_^2^* = 0.09, *ε* = 0.98, indicating that alerting modulated the alpha LPS flanker effect. Specifically, the alpha LPS flanker effect was significantly larger in the SOA 800 than in the SOA 100 condition, as indicated by the Flanker × Tone interaction in separate 2 × 2 ANOVA, *F*_1,34_ = 6.71, *p* = 0.014, *η_p_^2^* = 0.17. As seen in Figure 6A (lower panel), the flanker effect in the SOA 800 (dashed vs. solid blue lines) was notably larger than the flanker effect in the no-tone condition (dashed vs. solid black lines), mainly due to larger LPS in the incongruent condition (dashed blue line); although the Flanker × Tone interaction in the no-tone vs. SOA 800 comparison did not quite reach the significance threshold: *F*_1,34_ = 3.76, *p* = 0.061, *η_p_^2^* = 0.10. In the no-tone vs. SOA 100 comparison, the Flanker × Tone interaction was far from being significant. *F* < 1.0, n.s. The results therefore showed that here the alerting modulation was larger at 800 ms than 100 ms, and also numerically larger than no tone.

#### 3.5.2 Stimulus selection-related PCN (N2pc and SPCN)

Figure 6B depicts grand averages of the contralateral vs. ipsilateral ERP differences, relative to the target hemifield, from the PO7/8 sites. The difference waves show the posterior contralateral negativity (PCN) with its two large components: the N2pc, present 220–290 ms after target onset as the largest negative peak, followed by a slow wave of the SPCN emerging from about 320 ms.

The N2pc amplitude was smaller in the no-tone condition than in the two alerting conditions (see the upper panel of Figure 6B). This was confirmed by the main effect of Tone, *F*_1.76,59.78_ = 13.63, *p* < 0.001, *η_p_^2^* = 0.29, *ε* = 0.88. Comparison of the three tone conditions showed that all three differences between them were significant: no-tone vs. SOA 100: *F*_1,34_ = 8.80, *p* = 0.005, *η_p_^2^* = 0.21; no-tone vs. SOA 800: *F*_1,34_ = 23.09, *p* < 0.001, *η_p_^2^* = 0.40, and SOA 100 vs. 800: *F*_1,34_ = 5.65, *p* = 0.023, *η_p_^2^* = 0.14. The N2pc was significantly larger than zero in all three Tone conditions, *t*_34_ ≥ 10.36, *p* < 0.001. Other ANOVA effects were not significant, *F*s < 1.0, n.s., indicating no flanker effects on the N2pc.

The SPCN was significantly larger in the incongruent than in congruent flanker condition, *F*_1,34_ = 9.07, *p* = 0.005, *η_p_^2^* = 0.21 (see the lower panel of Figure 6B), which replicates our previous findings (Asanowicz et al., 2021, 2022). The SPCN was larger than zero in both flanker conditions, *t*_34_ ≥ 5.14, *p* < 0.001. Neither the main effect of Tone nor the Flanker by Tone interaction was significant, *F* ≤ 1.8, *p* ≥ 0.17, *η_p_^2^* ≤ 0.05, indicating no Tone effects on the SPCN.

### 3.6 Inter source phase coherence (ISPC)

#### 3.6.1 Midfrontal <−> lateral occipital (visual)

**Figure 7:**
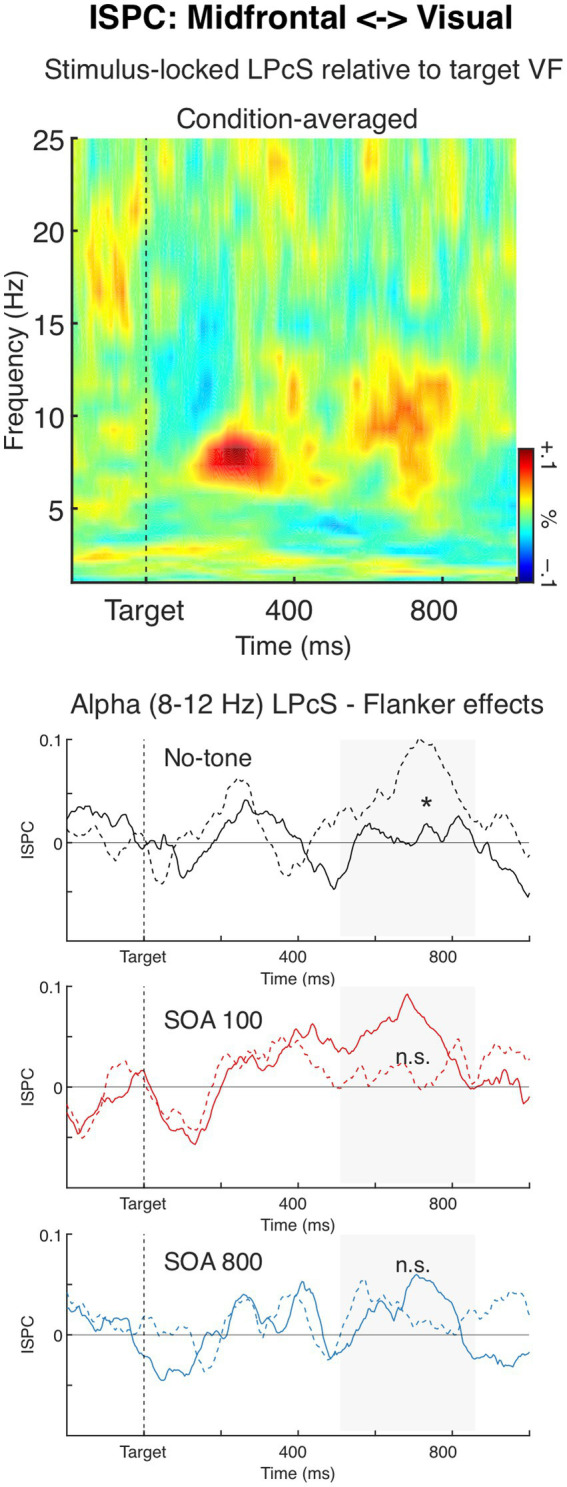
Inter Source Phase Coherence (ISPC) between the midfrontal and occipital (visual) sources. The time-frequency plot depicts condition-averaged lateralized phase coherence spectra (LP_C_S), relative to the target visual hemifield, for ISPC between the two sources. The LP_C_S result shows that ISPC was lateralized in the high theta (6–8 Hz) about 160–360 ms after target onset, and alpha (8–12 Hz) about 500–800 ms, indicating that theta and alpha ISPC were smaller at the contralateral than ipsilateral hemisphere. The charts below depict alpha-band LP_C_S over time, shown separately for the three Tone conditions, to illustrate the significant Flanker × Tone interaction (solid lines: congruent flankers; dashed lines: incongruent flankers). No significant effects of Flanker or Tone were observed for theta-band LP_C_S.

The time-frequency plot in Figure 7 shows condition-averaged lateralized phase coherence spectra (LP_C_S), relative to the target visual hemifield, for stimulus-locked ISPC between the midfrontal and occipital sources. As can be seen in the figure, ISPC was lateralized in the theta band (6–8 Hz) at about 160–360 ms after target onset, and in the alpha band (8–12 Hz) within 500–860 ms. Specifically, ISPC was modulated relatively to the target location so that theta and alpha coherence between the midfrontal and visual sources were generally stronger over the hemisphere ipsilateral than contralateral to the target hemifield. Both the theta and alpha ipsilateral vs. contralateral ISPC differences were significant, *t*_34_ = 4.66, *p* < 0.001, and *t*_34_ = 3.79, *p* < 0.001, respectively.

The ANOVA showed that Flanker and Tone had no impact on the theta-band LP_C_S, *F*s < 1.0, n.s. Whereas for the alpha-band LP_C_S there was a significant Flanker × Tone interaction *F*_1.94,66.10_ = 3.70, *p* = 0.031, *η_p_^2^* = 0.10, *ε* = 0.97. The interaction showed that alerting tone modified the flanker effect on the alpha LP_C_S, so that in the no-tone condition alpha LP_C_S was larger in the incongruent trials than in the congruent trials, *F*_1,34_ = 5.11, *p* = 0.030, *η_p_^2^* = 0.13 (see Figure 7), whereas in the other two Tone conditions the incongruent-congruent difference was not significant (SOA 100: *F*_1,34_ = 1.88, *p* = 0.18, *η_p_^2^* = 0.05; SOA 800 condition: *F* < 1.0, n.s.). No other significant effects were found, *F*s < 1.0.

#### 3.6.2 Midfrontal <−> centro-lateral (motor)

Stimulus-locked ISPC between the midfrontal and motor sources was notably lateralized relative to the responding hand in the alpha/mu band (8–13 Hz), with a peak at about 210 ms after target onset, and in the theta band (3–8 Hz), within a longer window approximately 450–900 ms after target onset. Both the alpha and theta lateralizations showed that ISPC was larger over the hemisphere contralateral to the responding hand than the ipsilateral one (indicated by negative LP_C_S values); *t*_34_ = 2.85, *p* = 0.007, and *t*_34_ = 3.28, *p* = 0.002, respectively. The alpha ISPC lateralization was stronger (i.e., alpha LP_C_S was more negative) in the incongruent than in the congruent flanker condition, *F*_1,34_ = 6.02, *p* = 0.019, *η_p_^2^* = 0.15. The main effect of Tone, and the Flanker × Tone interaction were not significant, *F* ≤ 1.9, *p* ≥ 0.16, *η_p_^2^* ≤ 0.05. The theta ISPC lateralization did not differ significantly between the Flanker and Tone conditions, *F* ≤ 2.2, *p* ≥ 0.15, *η_p_^2^* ≤ 0.06.

## 4 Discussion

### 4.1 Behavioral effects of alerting on conflict

The RT results showed the typical alerting effect on response conflict: while the responses were generally faster in the trials with alerting tone than in the no-tone trials, this speed gain was smaller in the trials with the incongruent flankers than in the congruent trials. Thereby the conflict score (incongruent *minus* congruent condition) was larger in alerting trials, which replicates the findings from numerous previous studies (e.g., Asanowicz et al., 2019; Böckler et al., 2011; Callejas et al., 2004, 2005; Fischer et al., 2012; Ishigami and Klein, 2010; Spagna et al., 2014).

The ERR results, however, did not show a significant alerting-conflict interaction. As mentioned in the introduction, Asanowicz and Marzecová (2017) observed that when the SOA interval between alerting stimulus and target onset was short (100 ms), the increased conflict-score in RTs was accompanied by also increased conflict score in ERR—indicating that phasic alerting worsened the performance. Whereas when the SOA was longer (400–800 ms), the increased conflict-score in RTs was accompanied by decreased conflict score in ERR—indicating that the extended conflict processing time was utilized for a more accurate conflict resolution. Following these findings, we introduced two SOA conditions in the current experiment: alerting tone was presented either 100 or 800 ms before target onset, which was aimed at capturing the alerting time-course. However, with the null interaction in ERR we were unable to confirm the hypothesis of the difference between the two SOA conditions. Possibly, as the overall accuracy was 96%, the current task was too easy to yield reliable alerting-related differences in error rates. Alternatively, the alerting time-course dynamics may be more subtle than we assumed, or context-dependent, warranting further investigation. Nevertheless, our EEG results did reveal interactive effects that point to a distinction between the two SOA conditions, suggesting that EEG measures may be more sensitive than behavioral error rates.

### 4.2 Electrophysiological activity

#### 4.2.1 Midfrontal activity

First of all, we observed that alerting increased conflict-related induced (i.e., time-locked but non-phased locked) midfrontal theta power (the effect was similar in both SOA conditions). This result confirms thereby the previous findings by Asanowicz et al. (2019) and Tromp et al. (2024). Importantly, alerting affected theta power predominantly in the incongruent flanker condition (in both SOA conditions), which suggests that the effect specifically reflects a modulation of conflict processing. Taking into account that increased conflict scores in the alerting conditions were also observed in RTs, the theta power effect supports the hypothesis that the stronger conflict in alerting trials (as shown in the RTs) increases the involvement of the neural mechanism dedicated to resolving this conflict.

Moreover, we observed that alerting modulated the midfrontal N2 component of the ERP (i.e., phase-locked activity). Interestingly, while conflict-related midfrontal theta power was larger in alerting conditions, the N2 was generally larger in no-tone condition (again, two SOA conditions did not differ between each other). In line with previous studies (e.g., Asanowicz et al., 2021; Cohen and Donner, 2013), these results suggest that midfrontal theta power and N2 tap into different aspects of processing, and, accordingly, are differently modulated by alerting. Taking into account their temporal dynamics, it might be that the N2 reflects a transient and relatively short-lived process of executive monitoring—that is, a signal indicating the need for action control; whereas the theta power modulation may reflect a longer-lasting process of conflict processing and resolution—that is, the implementation of executive control mechanisms to resolve the conflict (cf. e.g., Asanowicz et al., 2019, 2021; Cohen and Donner, 2013). Following these ideas, we may speculate that the N2 is larger in no-tone trials because the target appears without an accessory signal. Whereas in tone trials, the accessory signal may trigger proactive preparatory processes in advance, making the need for action control—and thus the N2 response—weaker.

We observed a similar N2 effect in our recent study on proactive control: when a cue provided advance information about the congruency of the upcoming trial, the N2 amplitude was reduced, compared to trials with a neutral uninformative cue (Asanowicz et al., 2022). The proposed interpretation corresponds with the interpretation of the so-called Gratton effect (Gratton et al., 1992; often referred to as the congruency sequence effect, cf. Egner, 2007) proposed within the conflict monitoring framework (Botvinick et al., 2001), which posits that the signals of conflict detection generated in the current conflict trial are more pronounced when the previous trial was a non-conflict trial, whereas the involvement of a conflict control mechanism in the current conflict trial is increased when the previous trials was also a conflict trial (see, e.g., Botvinick et al., 1999; Kerns, 2006). However, there is one caveat here to consider. Namely, unlike in many previous studies (including our own, Asanowicz et al., 2021, 2022), in the current study we did not observe a conflict-related modulation of the N2 component, but only a main effect of alerting. As noted in Results section, this absence is not entirely surprising, as the lack of a conflict-related N2 has been reported in previous studies (Asanowicz et al., 2019; Kałamała et al., 2018; Tillman and Wiens, 2011). It is unclear at the moment why the conflict effect on the N2 is absent in some studies. Notably, the task stimuli and procedure in the present study were very similar to those used in Asanowicz et al. (2021, 2022) where a conflict-related N2 increase was observed.

#### 4.2.2 Motor activity

We observed response-related lateralization of local power in the centro-lateral motor sources across the beta, alpha/mu, and theta bands. All of these response-related lateralizations of motor activity were modulated by flanker congruence. Namely, in the incongruent flanker condition the beta and mu LPS indices of lateralizations were larger—indicating a larger contralateral vs. ipsilateral power reduction, and the theta LPS was lower—indicating larger ipsilateral vs. contralateral power reduction, as compared to the congruent flanker condition. The modulations may reflect the effects of implementation of inhibitory control over selection and execution of the proper response within the motor cortex, which is obviously more demanding in the conflict trials. These results are in agreement with previous studies; we had observed flanker effects on response-related lateralization of beta and theta power in the motor areas in our previous experiments (Asanowicz et al., 2021; Panek et al., 2025; Van der Lubbe et al., 2025), and the involvement of beta and mu activity in motor control is well established in the literature (for a review see, e.g., Engel and Fries, 2010; Van Wijk, 2022).

Importantly, the flanker effect on the motor activity was modulated by alerting but only in the lateralization of mu power. Alerting tone presented 800 ms before target onset eliminated the congruent–incongruent difference on the response-related lateralization of local mu power, whereas in the SOA 100 condition the flanker effect on the mu lateralization was similar as in the no alerting condition. It is unclear to us at the moment on why the effect of alerting would appear only in the mu band while also the beta and theta activity were involved in response control. It is noteworthy however that this is our first neural indicator of a differentiation between the two SOA conditions (other were found in LRP latencies and lateralization of visual alpha power for, see below). According to our hypothesis, alerting tone with the SOA 800 ms would increase the efficiency of conflict processing due to endogenously increased readiness for processing incoming stimuli and better response preparation. We may therefore speculate that the longer preparation time available in the SOA 800 condition indeed facilitated preparation for the selection and activation of the proper response; so that a weaker conflict may have developed, and thus implementing response control only at a level similar to that in the congruent condition (as reflected in the lateralization of mu power) was sufficient for successful response execution.

Interesting results were observed in modulations of the latencies of stimulus-locked LRPs. First of all, the LRPs in the incongruent condition had typically longer latencies than LRPs in the congruent condition (cf. e.g., Asanowicz et al., 2019, 2021), corresponding with the flanker effect in RT. Second, as expected, the LPR latencies were generally shorter in the alerting trials than in the no-tone trials, which is consistent with the RT results (although the latencies were also slightly longer in the SOA 800 condition than SOA 100, which does not align with the RT data, where there was no difference between the two SOA conditions). Third, the flanker effect on the LRP latencies was modulated by alerting tone in the SOA 800 condition but not in the SOA 100 condition. That is, while the LRP flanker effect in the SOA 100 condition was similar to the no-tone condition, in the SOA 800 condition the LRP latency difference between congruent and incongruent trials increased, compared to the no-tone condition. This was because the alerting-related facilitation (i.e., latency decrease) in the SOA 800 condition (relative to the no-tone condition) was larger in the congruent trials than in the incongruent trials (see Figure 6A). This pattern again corresponds with the RT results. However, it remains unclear why such consistency with RTs was present in the SOA 800 but not in the SOA 100 condition. Importantly, the LRP latency results were consistent with the mu power results, both showing that alerting modulated local motor processes only in the longer SOA condition. Lastly, no effects of flanker and tone were observed in the response-locked LRPs, suggesting that all of these LRP latency modulations occurred before response execution, thus did not concern late motor processes (cf. Asanowicz et al., 2021; Hackley and Valle-Inclán, 1998).

#### 4.2.3 Visual activity

In terms of induced oscillatory activity, we observed target-related lateralization of local alpha power in the lateral occipital (visual) sources indicating a larger contralateral vs. ipsilateral alpha reduction (i.e., event-related desynchronization) beginning from about 350 ms after target onset (cf. Bacigalupo and Luck, 2019). As in our previous studies (Asanowicz et al., 2021, 2022), this contralateral alpha power reduction was larger and lasted longer in the incongruent than in the congruent trials. This shows that the effects of flanker-induced interference are present already in the visual processing, and it is consistent with the idea that stimulus selection and distractor suppression are parts of executive action control (Asanowicz et al., 2021; Verbruggen et al., 2014). Importantly, we also observed an interaction of this flanker effect on the alpha LPS index with alerting tone. Specifically, the alpha LPS flanker effect in the SOA 800 condition was larger than in the no-tone condition, and this was mainly due to larger LPS in the incongruent trials. No such difference was found in the comparison of the SOA 100 with the no-tone condition. As the effect stems from the modulation in the incongruent trials, we may speculate that it reflects a result of increased preemptive control of visual processing (cf. Asanowicz et al., 2022; Ridderinkhof et al., 2011; Verbruggen et al., 2014), likely enabled by an endogenous increase of alertness in the SOA 800 condition (cf. Asanowicz and Marzecová, 2017). The aim of this preemptive adjustment would be to prevent flanker interference by increasing the involvement of a visual suppression mechanism, presumably reflected in alpha power lateralization (cf. Klimesch, 2012; Jensen and Mazaheri, 2010).

An important differentiation between alerting and conflict processing was found in the target-related lateralizations of the visual ERPs. First, the N2pc component (Luck and Hillyard, 1990), which is thought to reflect target selection (Constant et al., 2025; Eimer, 1996), was larger in the alerting trials. There was also a small difference between the two alerting conditions (the N2pc was slightly larger in the SOA 800 condition). The results suggest that alerting amplifies attentional visual selection. This is in agreement with the hypotheses postulating that alerting modulates visual selection either by widening the scope of attention (Weinbach and Henik, 2011, 2012a, 2012b) or promoting spatial grouping (Schneider, 2018b), which in consequence increases the impact of incongruent flankers observed in the behavioral response times (cf. Introduction). The observed effect of alerting on the N2pc is also in line with previous findings showing that (i) alerting increased the amplitude of visual-evoked N1 potential, indicating a modulation of perceptual processing (Asanowicz et al., 2019), and (ii) alerting accelerated and enhanced the effects of pre-target preparatory spatial orienting, indicated in modulations of the early directing attention negativity (EDAN) and the late directing attention positivity (LDAP) (Asanowicz and Panek, 2020). Second, the SPCN (or the contralateral delay activity, Vogel and Machizawa, 2004), which follows the N2pc, was larger in the incongruent trials than in the congruent trials (as in Asanowicz et al., 2021, 2022). Unlike the N2pc, however, the SPCN was not modulated by alerting. As it has been suggested previously, this flanker congruency effect on the SPCN plausibly reflects “a process of creating and maintaining stable representations of the selected visual stimuli, which serves as the perceptual basis for processes of conflict resolution and the proper S-R integration” (Asanowicz et al., 2022, p. 1606; cf. Bacigalupo and Luck, 2019; Schneider et al., 2014).

#### 4.2.4 Functional connectivity

To assess functional connectivity of the midfrontal area with the lateral visual and motor areas, we computed ISPC between the sources and then LP_C_S relative to the target visual hemifield and the responding hand, respectively. A target location-related lateralization of connectivity with the visual sources was observed in the theta and alpha bands. The lateralization showed theta and alpha phase coherence between the midfrontal and visual sources were generally stronger over the hemisphere ipsilateral than contralateral to the target location. These modulations are presumably related to inhibitory signals triggering suppression of the processing of irrelevant distractors in the hemifield opposite to the target location (Asanowicz et al., 2023; Van der Lubbe et al., 2023, 2025). Importantly, the lateralization of alpha ISPC was affected by both flanker congruency and alerting. In the no-tone trials, the lateralization of alpha ISPC was stronger in the incongruent than in the congruent condition, indicating a stronger inhibition of the irrelevant distractors in the conflict trials (note that in the current task an array of distractors was simultaneously presented in the hemifield opposite to the target location). Whereas in the alerting trials (with both SOAs) this flanker effect was not present anymore. The flanker effect (in the no-tone trials) on the relatively late target/distractors-related connectivity may reflect a stronger reliance on post-target stimulus-driven, i.e., reactive, implementation of conflict control in the conflict trials, as in this case the target was not signaled by any accessory stimulus. Whereas in the two alerting conditions, the tone could work as an accessory signal allowing for some processes to be mobilized already at target onset, thereby diminishing demands for reactive inhibitory control. However, if this interpretation holds true, then—according to our hypothesis—the alerting effect should be stronger for SOA 800 than for SOA 100, which we did not observe. This warrants further research.

A response-related lateralization of connectivity between the midfrontal and motor sources was present in the alpha/mu and theta bands. Both indicated a larger ISPC increase over the hemisphere contralateral to the responding hand than the ipsilateral hemisphere. Moreover, the alpha-band connectivity lateralization was stronger in the incongruent than in the congruent flanker condition. As suggested by Van der Lubbe et al. (2025), these connections may play a role in releasing inhibition for the correct response program, enabling its execution. Here, however, we did not observe any statistically significant effects of alerting.

### 4.3 Alerting vs. temporal preparation and temporal orienting

Using the two tone-target SOA intervals (100 and 800 ms), we aimed to differentiate between the effects of fast but short-lived exogenous alerting and slower but longer lasting endogenous alerting. As mentioned in the introduction, endogenous alertness is related to a “top-down” increase of expectancy and readiness, which prepares sensory and motor systems to deal with the expected upcoming stimulus (cf. e.g., Fan et al., 2007; Klein and Ivanoff, 2011; Hackley et al., 2009). However, this raises the issue of a potential confound between phasic alerting and foreperiod-based^4^ temporal expectancy, temporal preparation, and attentional temporal orienting (cf. e.g., Hackley et al., 2009; Weinbach and Henik, 2012b, 2013). This pertains not only to definitions and methodology/operationalizations, but also to the underlying brain mechanisms (reflected, e.g., in the contingent negative variation, or CNV, component of the ERP; see, e.g., Brunia et al., 2012).

Temporal orienting refers to the ability to endogenously direct attention to a particular moment in time based on available information, e.g., a cue presented prior to target onset (Coull and Nobre, 1998; Correa, 2010). Whereas temporal preparation may be understood as a broader phenomenon, as it may also include a less time-specific preparation of sensory and cognitive systems, which makes it even harder to distinguish from alerting. In the alerting tasks, temporal orienting will be involved particularly when the SOA interval is constant (like in the standard ANT procedure where the SOA is 500 ms, Fan et al., 2002), as in this case the alerting stimulus predicts the exact target onset. In the current study—which used two SOA intervals randomized trial-wise—when the target did not appear within the short foreperiod (SOA 100), its onset in the long foreperiod (SOA 800) became certain. Consequently, temporal preparation should increase as time elapsed, possibly also engaging temporal orienting in the long SOA condition (cf. Weinbach and Henik, 2012b). Moreover, even if the alerting signal were not to allow for specific temporal prediction of the target onset (cf. e.g., Lawrence and Klein, 2013), thereby making it impossible to direct attention to the exact moment, it would still trigger some temporal preparation simply by signaling the rapid target appearance. Yet another aspect of this issue is that a warning signal may simultaneously trigger both alertness and non-specific motor preparation (non-specific because neither the target condition nor the required response can yet be predicted), and both may interact with response conflict resolution.

Experimental evidence on this matter remains limited and inconclusive. For instance, while a study by Weinbach and Henik (2013) demonstrated that the alerting-conflict interaction could not be explained by temporal expectancy, Correa et al. (2010) reported contradictory evidence showing that temporal orienting increased flanker conflict, much like phasic alerting. Importantly, in an EEG/EMG study using the Simon task, Korolczuk et al. (2022) observed that in conflict trials, motor cortex inhibition of the incorrect hand was weaker for temporally predictable targets. In other words, when the target stimulus triggered two conflicting responses, temporal predictability reduced the suppression of the improper response. Obviously, more empirical evidence is needed, along with further methodological and theoretical developments on this issue.

## 5 Concluding remarks

To summarize, the effects of alerting that either did not differ between the SOA 100 and SOA 800 conditions, or differed only slightly and were significant for both, were observed in the current study as:

(i) an increase in conflict-related midfrontal theta power (no difference between SOAs),
(ii) a decrease in midfrontal N2 amplitude (no difference between SOAs),
(iii) a decrease in LRP latency (slightly larger effect for SOA 100),
(iv) an increase in N2pc amplitude (slightly larger effect for SOA 800).

In contrast, the effects that appear specific for endogenous alertness, i.e., those present only in the SOA 800 condition, were observed as:

(i) a suppression of the flanker effect on response-related lateralization of alpha/mu power (which was otherwise present in both the no-tone and SOA 100 conditions),
(ii) an increase in the flanker effect on LRP latency,
(iii) an increase in the flanker effect on the target-related contralateral suppression of visual alpha power.

Consistent with previous studies (see Introduction), the present findings demonstrate that alerting impacts task-relevant processing at multiple stages—from target selection, through central executive S-R integration, to the selection and activation of the proper motor response—and in several distinct ways (e.g., interacting with the flanker effects either directly or indirectly). This indicates that alerting affects both conflict emergence and conflict resolution processes. The observation that alerting effects appeared in most of the analyzed task-related neural modulations (and in all isolated sources) suggests that these are not focal effects confined to specialized brain regions, but rather network-level interactions with dynamic involvement of the nodes essential for a given process at the moment. Such a functional network may constitute an effective “selection-for-action” system (Asanowicz et al., 2021; Allport, 1987), in which the medial frontal cortex may work as an “executive hub” coordinating the processes of dynamic coupling and decoupling of currently relevant perceptual and motor information into sensorimotor schemas or “event-files” (cf. Cavanagh and Frank, 2014; Cisek and Kalaska, 2010; Frings et al., 2020). Alerting—considered both as a function and as a neural network—may act as a key modulator of the neural dynamics underlying the functioning of the selection-for-action system.

## Data Availability

The raw data supporting the conclusions of this article will be made available by the authors, on request.

## Footnotes

^1^ www.psychopy.org

^2^ www.biosemi.com/faq/cms&drl.htm

^3^Task-related modulations of the EEG signal reflect a combination of evoked and induced activity (see, e.g., Cohen, 2014b; David et al., 2006). Evoked activity is phase-locked to the onset of an event, while induced activity is time-locked but not phase-locked. The precise cognitive processes reflected by these two types of activity remain a matter of debate. Here, we extracted induced power to ensure that the time–frequency and ERP analyses included non-overlapping signal components.

^4^Foreperiod is the time interval between a warning signal and the target.
